# The role of quiescent thymic progenitors in TAL/LMO2-induced T-ALL chemotolerance

**DOI:** 10.1038/s41375-024-02232-8

**Published:** 2024-03-29

**Authors:** Kevin W. O’Connor, Kensei Kishimoto, Irena O. Kuzma, Kelsey P. Wagner, Jonathan S. Selway, Justine E. Roderick, Keshab K. Karna, Kayleigh M. Gallagher, Kai Hu, Haibo Liu, Rui Li, Michael A. Brehm, Lihua Julie Zhu, David J. Curtis, Cedric S. Tremblay, Michelle A. Kelliher

**Affiliations:** 1 Department of Molecular, Cell and Cancer Biology, University of Massachusetts Chan Medical School, Worcester, MA 01605 USA; 2 Medical Scientist Training Program, University of Massachusetts Chan Medical School, Worcester, MA 01605 USA; 3 Program in Molecular Medicine, University of Massachusetts Chan Medical School, Worcester, MA 01605 USA; 4 Australian Centre for Blood Diseases (ACBD), Central Clinical School, Monash University, Melbourne, VIC 3004 Australia; 5 Department of Immunology, Rady Faculty of Health Sciences, University of Manitoba, Winnipeg, MB R3E 0T5 Canada; 6 Paul Albrechtsen Research Institute CCMB, CancerCare Manitoba (CCMB), Winnipeg, MB R3E 0V9 Canada

**Keywords:** Cancer models, Cancer stem cells, Oncogenes, Chemotherapy, Acute lymphocytic leukaemia

## Abstract

Relapse in T-cell acute lymphoblastic leukemia (T-ALL) may signify the persistence of leukemia-initiating cells (L-ICs). Ectopic TAL1/LMO expression defines the largest subset of T-ALL, but its role in leukemic transformation and its impact on relapse-driving L-ICs remain poorly understood. In *TAL1*/*LMO* mouse models, double negative-3 (DN3; CD4^−^CD8^−^CD25^+^CD44^−^) thymic progenitors harbored L-ICs. However, only a subset of DN3 leukemic cells exhibited L-IC activity, and studies linking L-ICs and chemotolerance are needed. To investigate L-IC heterogeneity, we used mouse models and applied single-cell RNA-sequencing and nucleosome labeling techniques in vivo. We identified a DN3 subpopulation with a cell cycle–restricted profile and heightened *TAL1/LMO2* activity, that expressed genes associated with stemness and quiescence. This dormant DN3 subset progressively expanded throughout leukemogenesis, displaying intrinsic chemotolerance and enrichment in genes linked to minimal residual disease. Examination of *TAL*/*LMO* patient samples revealed a similar pattern in CD7^+^CD1a^−^ thymic progenitors, previously recognized for their L-IC activity, demonstrating cell cycle restriction and chemotolerance. Our findings substantiate the emergence of dormant, chemotolerant L-ICs during leukemogenesis, and demonstrate that *Tal1* and *Lmo2* cooperate to promote DN3 quiescence during the transformation process. This study provides a deeper understanding of *TAL1/LMO*-induced T-ALL and its clinical implications in therapy failure.

## Introduction

Current therapeutic strategies for T-cell acute lymphoblastic leukemia (T-ALL) involve high-dose multi-agent regimens that incorporate chemotherapeutic agents (e.g., vincristine and L-asparaginase) alongside glucocorticoids (e.g., dexamethasone) [1]. Despite optimization of these intensified treatment protocols, approximately 25% of pediatric T-ALL patients face therapy-refractory or relapsed disease, representing a major barrier to successful management [2]. The prevailing hypothesis implicates leukemia-initiating cells (L-ICs) as the relapse-inducing culprits. These cells are present at diagnosis but are often quiescent, rendering them resistant to cytotoxic agents that target actively proliferating cells [3]. However, conclusive evidence linking dormant L-ICs to chemotolerance and relapse is needed.

L-IC populations have been documented in T-ALL patients [4–6] and mouse models [7–10]. While acute myeloid leukemia studies have established the CD34^+^CD38^−^ hematopoietic stem and progenitor cell population as harboring L-ICs [11], defining a similar cellular hierarchy in T-ALL has proven challenging [12–14]. Mouse models have significantly enhanced our understanding of L-IC biology in T-ALL, particularly the clinically relevant *Tal1/Lmo2* transgenic mouse model [15]. Ectopic expression of TAL1, a basic helix-loop-helix transcription factor, defines the largest molecular subgroup of T-ALL patients [16]. Transgenic *Tal1* expression in thymocytes induces T-ALL in mice, a process accelerated by co-expression with its binding partners, *Lmo1* or *Lmo2* [7, 15, 17, 18]. Transgenic expression of *Tal1* disrupts thymocyte development by interfering with E47/HEB-dependent gene expression, resulting in an accumulation of CD4^−^CD8^−^ double-negative (DN) thymocytes [8, 15, 19]. Limiting dilution assays have demonstrated that double-negative 3 (DN3; CD4^−^CD8^−^CD25^+^CD44^−^), but not CD4^+^CD8^+^ double-positive (DP) cells, exhibit L-IC activity, however, estimates of L-IC frequency suggest that they are indeed a rare subset within the DN3 cell population in T-ALL [7, 8].

Elucidation of L-IC gene expression signatures may reveal L-IC-specific genes and pathways that can be targeted to prevent relapse. We employed a combination of single-cell RNA-sequencing (scRNA-seq) and in vivo nucleosome labeling techniques in a mouse model to unveil previously unobserved heterogeneity within the L-IC-enriched DN3 population. Within this population, we identified a subpopulation of “dormant” DN3 leukemic cells marked by nucleosome label retention, indicative of reduced cell cycling. These cells possessed transcriptional attributes associated with stemness, inflammation, and quiescence. Our investigations demonstrate that, throughout leukemogenesis, *Tal1* and *Lmo2* act in synergy to promote DN3 quiescence and chemotolerance. Importantly, dormant DN3 (dDN3) cells displayed a transcriptional signature akin to that found in dormant ALL patient cells and minimal residual disease (MRD) ALL samples, highlighting the clinical relevance of this mouse L-IC subpopulation. In alignment with these findings, human CD7^+^CD1a^−^ L-ICs (hL-ICs) from T-ALL patients likewise exhibit the TAL/LMO gene expression program, cell cycle restriction and chemoresistance, implying a shared phenotype between mouse and human L-ICs.

## Materials and methods

### scRNA-seq analysis

All mouse experiments were approved by the UMass Chan Institutional Animal Care and Use Committee. Thymi from 6-week-old littermate wild-type (WT), preleukemic, or leukemic proximal *Lck-Tal1/Lmo2* mice (FVB/N) [15] were mechanically dissociated, and RBCs were lysed using ammonium-chloride-potassium (ACK) buffer. Cells were resuspended in phosphate-buffered saline (GibcoThermoFisher, USA). Viable (7-AAD^−^) singlets were sorted via fluorescence-activated cell sorting and loaded onto the Chromium Controller (10X Genomics, 3’v3 and v3.1 Pleasanton, CA) to achieve a desired yield of 10^4^ cells per sample.

Single-cell libraries were prepared according to 10X Genomics instructions. cDNA and Illumina libraries were quality-checked by Advanced Analytic Fragment Analyzer (UMass Chan Molecular Biology Core Laboratory) and subsequently sequenced on a HiSeq platform with paired-end sequencing (2 × 150 bp) performed by Genewiz/Azenta Life Sciences (Burlington, MA). FASTQ files were processed using the Dolphin Next platform [20], and further analyses were conducted using the Seurat package [21] in R (v4.0).

For additional details, please see Supplementary Methods.

### Label retention studies

6-week WT and Proximal *Lck*-*Tal1, Lmo2*, or *Tal1/Lmo2* C57B6/J mice [22] were utilized. Mice were engineered with heterozygous expression of *Col1a1*-*H2B-GFP* and *Rosa26* reverse tetracycline activator reporter alleles [23]. A 6-week doxycycline pulse was administered, followed by a 2-week chase period, culminating in the harvest of thymi from 14-week-old mice.

Doxycycline (2 g/l; Sigma Livonia, MI) was administered in drinking water supplemented with sucrose (10 g/l); medicated water was changed twice per week. Thymi from pulse-chased mice were mechanically dissociated into a single-cell suspension, ACK-lysed, and resuspended in PBS in preparation for immunostaining.

### In vivo BrdU incorporation

NOD scid gamma (NSG) mice harboring patient-derived xenograft (PDX) cells were administered 1 mg of BrdU (APC BrdU (BD Pharmingen, Kit #552598 Torrey-Pines, CA)) via intraperitoneal injection 1 h before sacrifice. Bone marrow and spleen were processed as outlined in Supplementary Methods, followed by immunostaining. Subsequently, cells were fixed and permeabilized according to the manufacturer’s instructions, treated with DNAse to expose the incorporated BrdU, stained with anti-BrdU-APC antibody, counterstained with 7-AAD, and analyzed by flow cytometry.

## Results

### Notch1 dysregulation and thymocyte progenitor accumulation in T-ALL revealed by scRNA-seq

**Fig. 1:**
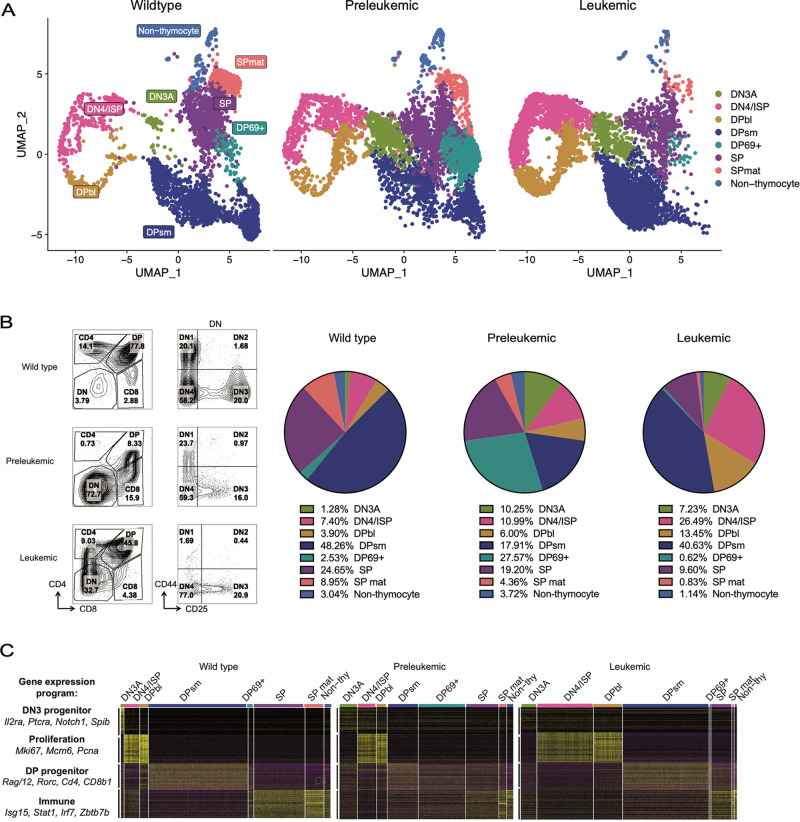
scRNA-seq captures developmental perturbations associated with TAL1/LMO-mediated leukemogenesis. **A** Top panel, integrated UMAP clustering of wild type (WT), preleukemic (PL) and leukemic thymi. Clusters are annotated based on SingleR predictions using differentially expressed genes (DEGs) in WT. Bottom panel, pie charts showing distribution of WT, Preleukemic, and Leukemic cells in each cluster. DN3a = double-negative 3a, DN4/ISP = double-negative 4/immature single-positive, DPbl = CD4 and CD8 double-positive blast, DPsm = double-positive DP small resting, DP69+ = double-positive post-selection CD69^+^, SP = CD4 single-positive and CD8 single-positive, SPmat = CD4 single-positive and CD8 single-positive SP, “mature” and non-thymocyte. **B** Immunophenotype of WT (6-week), *Tal1/Lmo2* preleukemic (6-week) thymus, and *Tal1/Lmo2* leukemia (3 month) analyzed by scRNA-seq. Right panels are gated on CD4^-^CD8^-^ (DN) cells. **C** Heatmap of consensus non-negative matrix-factorization (cNMF) analysis showing the top 100 differentially expressed genes between WT cNMFs plotted for WT, PL, and leukemic samples. Gene expression programs identified by cNMF analysis are shown on the left. Integrated cluster annotations are shown above.

To profile transcriptional changes that accompany leukemic transformation with single-cell resolution, we performed scRNA-seq on WT, preleukemic, or leukemic *Tal1/Lmo2* thymi. Aggregated single cells were then analyzed using integrated uniform manifold approximation and projection (UMAP) clustering [21]. Using WT as the reference, we annotated the resultant eight clusters as double-negative 3a (DN3a), double-negative 4(DN4)/immature single-positive (ISP), CD4 and CD8 double-positive blast (DPbl), double-positive DP small resting (DPsm), double-positive post-selection CD69^+^ (DP69^+^), single-positive (SP), SP mature (SP mat) and non-thymocyte (Fig. 1A, Supplementary Fig. 1A–C, Supplementary Table 1). Concordant with immunophenotype analysis (Fig. 1B) and our previous studies [8, 15], we observed expanded DN3a and DN4/ISP populations and decreased SP/SP mat cells in preleukemic and leukemic thymi (Fig. 1A, B).

To more finely dissect how thymocyte development is perturbed during leukemogenesis, we performed consensus non-negative matrix factorization, a method to infer cellular identity and cell type-independent (cell activity) gene expression programs [24], on WT thymocytes. The top four factorizations reliably captured normal developmental-stage gene expression programs: DN3 progenitor, expressed in DN3a cells; Proliferation, expressed in DN4/ISP and DPbl cells; DP progenitor, expressed in DPbl cells as they transitioned to DPsm cells; and Immune, expressed highly in SP and SP mat cells with lower expression in DN4/ISP and DP69^+^ clusters (Fig. 1C, Supplementary Fig. 1C). Strikingly, both preleukemic and leukemic cells maintained the DN3 progenitor program across clusters, including DP and SP cells (Fig. 1C, Supplementary Fig. 1E). This likely reflects inappropriate Notch1 activation, as Notch1 target genes were highly expressed in leukemic cells compared to WT and preleukemic cells, which persisted across all developmental stages (Supplementary Fig. 1E, F).

Preleukemic thymocytes showed diminished *Cd4*, *Cd8a*, and *Ptcra* mRNA expression and reduced CD4 surface expression (Supplementary Fig. 1F, Fig. 1B), consistent with TAL1-mediated E47/HEB inhibition of CD4 expression [19]. Surprisingly, preleukemic cells were still detected in DPsm and DP69^+^ clusters, albeit with diminished expression of the DP progenitor program, which also coincided with inappropriate expression of the immune program in these clusters, indicating transcriptional dysregulation in the preleukemic thymus (Fig. 1C). While the preleukemic stage showed an intermixing of DP progenitor and immune transcriptional programs, leukemic transformation coincided with the expansion of the DN4/ISP and DPbl at the expense of SP and SP mature thymocytes (Fig. 1B–C).

### Single-cell transcriptional profiling reveals dormant and proliferative DN3 clusters in preleukemic and leukemic mice

We next utilized scRNA-seq to uncover transcriptional heterogeneity among the L-IC-containing DN3 population within the context of *Tal1/Lmo2-*induced primary mouse leukemias, T-ALL#1 (Fig. 1B) and T-ALL#2 (Supplementary Fig. 2A). Both leukemia samples exhibited expression of active/cleaved NOTCH1, which responded to gamma-secretase inhibitor treatment (Supplementary Fig. 2B).

**Fig. 2:**
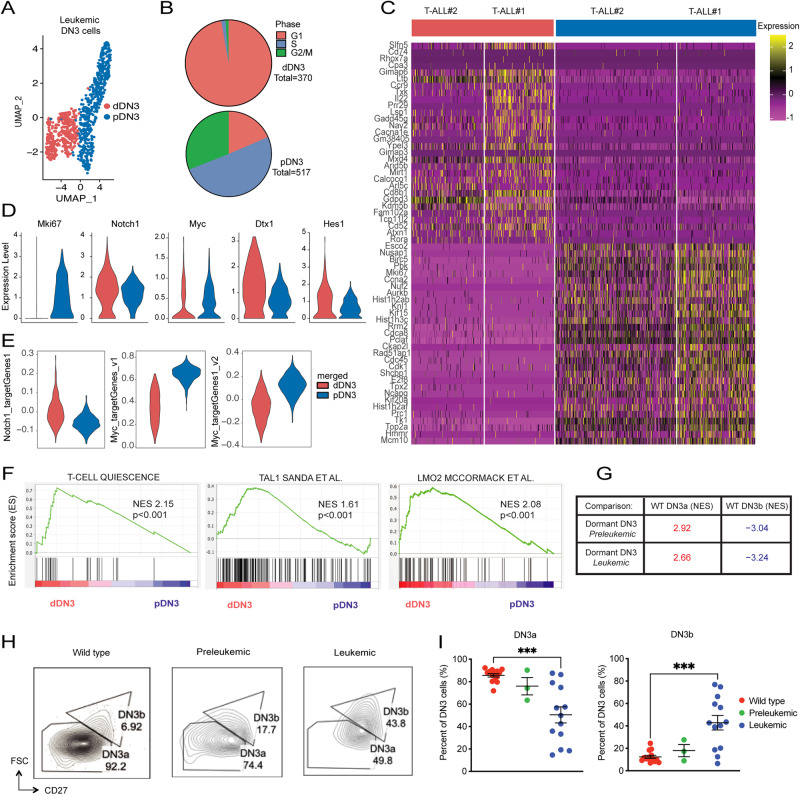
Identification of dormant and proliferative leukemic DN3 subpopulations during leukemogenesis. **A** Integrated UMAP clustering of T-ALL#1 and T-ALL#2 DN3 cells showing the dormant (dDN3) (red) and proliferative (pDN3) (blue) clusters. **B** Seurat cell cycle annotation of individual cells from dDN3 and pDN3 clusters for T-ALL#1 and T-ALL#2. **C** Heatmap of top 30 DEGs between dDN3 and pDN3 clusters. **D** Violin plots showing log2-transformed mRNA expression of indicated genes in dDN3 (red) and pDN3 (blue) T-ALL cells. **E** Violin plots showing Notch1, Myc v1, and Myc v2 module scores for dDN3 and pDN3 clusters analyzed by Wilcoxon rank-sum test, *p* < 2.2e-16. **F** GSEA using published data sets of T-cell quiescence (Eltanbouly et al. Science, 2020) and TAL1 (Sanda et al. *Cancer Cell*, 2012) and LMO2 target genes (McCormack et al. *Science*, 2010). **G** Summary of GSEA comparisons between preleukemic and leukemic dDN3 and pDN3 signatures and WT DN3a and DN3b gene sets (*p* < 0.001 for all comparisons). **H** Representative flow cytometry plots of DN3a (FSC^LO^, CD27^LO^) and DN3b (FSC^HI^, CD27^HI^) subpopulations. Plots are gated on DN3 (CD4^-^CD8^-^CD25^+^CD44^-^) cells^.^ **I** Quantification of DN3a and DN3b subpopulations in WT (*n* = 14), *Tal1/Lmo2* preleukemic (*n* = 3), or *Tal1/Lmo2* leukemic (*n* = 13) thymi. Data shown as percentage of all DN3 cells.

Leukemic DN3 cells from combined leukemic samples were selected based on lineage markers, integrated, and reclustered (Fig. 2A, Supplementary Methods). This analysis revealed two distinct transcriptional states, termed dormant DN3 (dDN3) and proliferative DN3 (pDN3), according to their differential expression of proliferation and developmental stage-associated genes (Fig. 2A). Cell cycle analysis (Seurat) of the integrated leukemic DN3 clusters confirmed that the majority of dDN3 cells were in G0/G1 phase (97%), while the majority of pDN3 cells were in S and G2/M phases (81%, Fig. 2B).

Compared to pDN3 cells, dDN3 cells exhibited higher expression of *Notch1* and Notch1-target genes, including *Dtx1* and *Hes1*, with lower expression of the proliferation-associated antigen *Mki67* (Fig. 2C–E). Interestingly, despite high *Notch1* expression and activity in dDN3 cells, there was reduced *Myc* expression and activity (Fig. 2D, E). Gene Set Enrichment Analysis (GSEA) showed enrichment for T-cell quiescence genes [25] (*Txnip*, *Btg1*, *Trib2*, *Ccr7*, *Btg2*, *Klf6*, *Pnrc1*, *Zfp36*, and *Klf2*), as well as TAL1 (*Arid5b, Cd53, Btg1, Gimap4*, and *Trib2*) and LMO2 (*Cpa3*, *Gimap6, Hhex, Btg1*, and *Gimap7*) signatures, in comparison to pDN3 cells (Fig. 2F) [9, 26]. Furthermore, dDN3 cells were enriched for stress- and inflammation-related pathways, while pDN3 cells were enriched for the MYC pathway, proliferation, DNA repair, and metabolism genes (Supplementary Table 1).

Analysis of preleukemic DN3 thymocytes also revealed dDN3 and pDN3 clusters, along with a third cluster identified as γδ T cells by SingleR (Supplementary Fig. 2C–E). Consistent with published work showing that low Notch1 expression favors γδ T-cell lineage choice [27], the γδ thymocyte cluster expressed lower *Notch1* and *Myc* levels compared to the dDN3 and pDN3 clusters and showed low *Mki67* expression (Supplementary Fig. 2D). The γδ cluster expressed high levels of *Ccr9*, *Cd52*, *Rora*, *Cd226*, and *Il18r1*, which were not detected in the dDN3 or pDN3 clusters (Supplementary Fig. 2D, E). Cell surface staining did not detect an increase in γδ thymocytes in the preleukemic thymus (Supplementary Fig. 2F), suggesting that the expression of γδ gene segments in preleukemic DN3 progenitors reflects germline transcription of these genes [28, 29].

Analysis of preleukemic dDN3 cells revealed high expression of Notch1, but low *Myc* mRNA and Myc-target gene expression (Supplementary Fig. 2D), similar to leukemic subpopulations (Fig. 2D, E). GSEA comparing dDN3 and pDN3 clusters also showed an enrichment of T-cell quiescence genes and TAL1 and LMO2 target genes (Supplementary Fig. 2G), suggesting that similar DN3 transcriptional states exist during the preleukemic and leukemic stages.

To determine whether dDN3 cells resemble quiescent DN3a stage thymocytes, we generated DN3a and DN3b signatures from single WT DN3 thymocytes (Supplementary Fig. 3A–D). Comparing the pseudobulked transcriptomes of dDN3 and pDN3 preleukemic and leukemic cells to WT DN3a and DN3b signatures revealed enrichment of the DN3a signature in the dDN3 cluster and enrichment of the DN3b signature in the pDN3 cluster (Fig. 2G). To account for the possibility that these findings were solely due to proliferative differences between the two subpopulations, we excluded cell cycle–associated genes from the gene sets [30]. Re-analysis following exclusion of cell cycle–related genes did not affect enrichment of the respective WT DN3a and DN3b signatures, suggesting that dDN3 and pDN3 leukemic cells maintain a shared gene expression program with their corresponding thymocyte developmental stage (Supplementary Fig. 3E). Immunostaining confirmed the presence of *Tal1/Lmo2* preleukemic and leukemic DN3a (FSC-A^LO^, CD27^LO^) and DN3b (FSC-A^HI^, CD27^HI^) cells, present in nearly equal proportions to the dDN3 and pDN3 clusters, respectively (Fig. 2H, I) [31].

### *TAL1* and *LMO2* promote DN3 thymocyte quiescence during leukemogenesis

Our scRNA-seq data suggest that there is a subset of cell cycle–restricted DN3 cells during leukemogenesis. To validate the cell cycle kinetics of DN3 thymocytes, we utilized the pulse-chase nucleosome labeling method, previously used to detect and isolate hematopoietic stem cells and preleukemic stem cells [23, 32]. We employed a tetracycline-inducible histone 2B-GFP (H2B-GFP) fusion protein that is incorporated into nucleosomes and subsequently used to discriminate between quiescent cells that retain H2B-GFP-label following withdrawal of doxycycline and cells that divide and thereby dilute the H2B-GFP label accordingly.

**Fig. 3:**
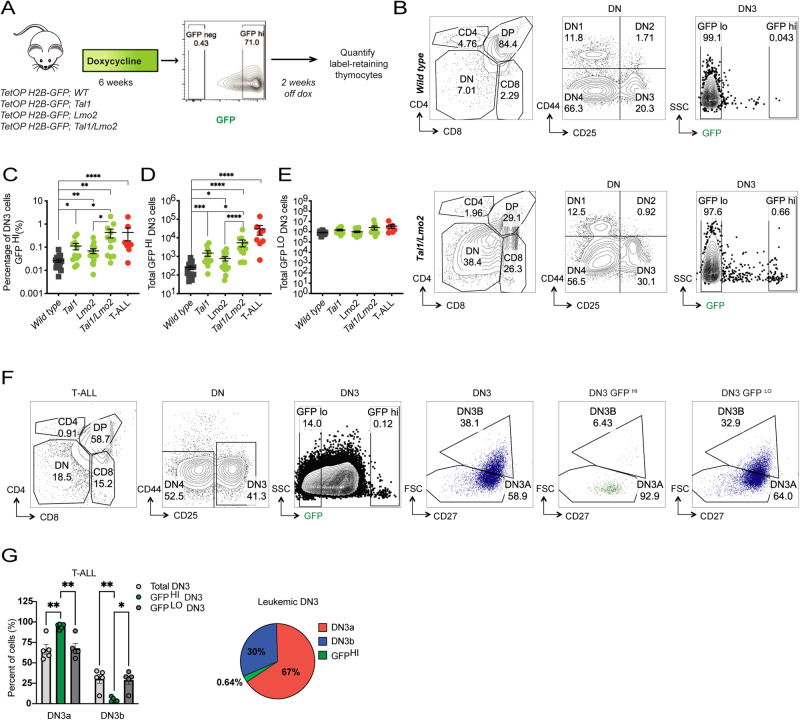
Tal1 and Lmo2 promote DN3 label retention during leukemogenesis. **A** Design of in vivo label retention studies using the doxycycline-inducible H2B-GFP mouse reporter. **B** Representative flow cytometry plots of WT (top row) and preleukemic *Tal1/Lmo2* (bottom row) thymi after pulse-chase. Left column, gated on single cells, middle column gated on DN, right column gated on DN3 cells. Quantification of (**C**), percentage of DN3 cells scored as GFP^HI^ after the 2-week chase period (**D**), total DN3 GFP^HI^ thymocytes and (**E**), total GFP^LO^ DN3 thymocytes in WT (*n* = 17; gray), preleukemic *Tal1* (*n* = 11; green), preleukemic *Lmo2* (*n* = 16; green), preleukemic *Tal1*/*Lmo2* (*n* = 12; green) mice, or leukemic mice chased for 2–3 weeks (T-ALL; *n* = 8; red). **F** Representative FACS plots of T-ALL cells after pulse-chase showing co-staining for DN3a/DN3b subpopulations. From left to right, cells were gated on singlets, DN, total DN3 cells, DN3 GFP^HI^ and DN3 GFP^LO^. First DN3 plot gated shows label retention of all DN3 cells and second plot shows DN3a/b distribution among DN3 cells. Gating on DN3 GFP^HI^ and DN3 GFP^LO^ shows that dormant cells are significantly enriched among the DN3a subpopulation. **G** Left panel, quantification of the percentage of total, GFP^HI^ or GFP^LO^ leukemic DN3 cells that scored as DN3a or DN3b (*n* = 5). Right panel, pie chart showing the relative frequencies of each leukemic subpopulation, with GFP^HI^ cells comprising a minor fraction (0.64%) of the DN3a subpopulation. All error bars represent mean $\pm \,$SEM. *p < 0.05, **p < 0.01, ***p < 0.001, ****p < 0.0001.

We treated 6-week WT or leukemia-prone *Tal1, Lmo2*, and *Tal1/Lmo2* mice expressing the reporter with doxycycline for 6 weeks to induce H2B-GFP expression (Fig. 3A). Following the pulse period, flow cytometry analysis revealed that the majority of DN3 thymocytes were GFP^HI^, indicating successful incorporation of H2B-GFP (Fig. 3A). However, a minority of cells remained GFP negative, suggesting that they did not divide and failed to incorporate the H2B-GFP during the pulse period.

Two weeks after discontinuing the doxycycline treatment (age 14-weeks), most thymocyte progenitors were found in the GFP^LO^ gate, indicating their division and the gradual loss of H2B-GFP from their nucleosomes (Fig. 3B). Consistent with their quiescent nature [32], 3% of DN1 thymocytes remained GFP^HI^ after the chase period, and there was no significant difference between WT and preleukemic mice (Supplementary Fig. 4A).

Focusing on the L-IC-containing DN3 progenitors, we observed an increased frequency and absolute number of GFP^HI^ DN3 cells in preleukemic *Lmo2* mice compared to WT mice (Fig. 3C, D), consistent with a recent study utilizing the *Cd2-Lmo2* transgenic model [32]. Additionally, we observed an increase in GFP^HI^ DN3 cells in preleukemic *Tal1* mice, indicating that *Tal1* also promotes cell cycle restriction in DN3 thymocytes (Fig. 3C, D). *Tal1/Lmo2* mice exhibited the highest frequency and absolute number of GFP^HI^ DN3 cells (Fig. 3C, D), suggesting that *Tal1* and *Lmo2* cooperate to promote DN3 quiescence during leukemogenesis.

Consistent with our previous work [15, 17, 19], we observed a significant reduction in preleukemic *Tal1* or *Tal1/Lmo2* thymic cellularity compared to WT mice (Supplementary Fig. 4B). Despite this finding, *Tal1* and *Tal1/Lmo2 mice* displayed 6- and 21-fold increases, respectively, in the absolute numbers of GFP^HI^ DN3 cells (Fig. 3D). Notably, there were no significant differences in the number of GFP^LO^ DN3 cells between genotypes (Fig. 3E), indicating that Tal1 and Lmo2 do not stimulate thymocyte proliferation during the preleukemic stage. Furthermore, we detected an increased frequency of GFP^HI^ cells in preleukemic DN4 and DP thymocytes from *Tal1* and *Tal1/Lmo2*, but not *Lmo2*-only mice, suggesting that *Tal1* promotes label retention of other progenitor populations (Supplementary Fig. 4A).

At the end of the pulse-chase period, a subset of *Tal1* or *Tal1/Lmo2* mice developed leukemia. These leukemic mice showed a similar frequency of GFP^HI^ DN3 cells compared to preleukemic *Tal1/Lmo2* mice, with the highest absolute number of GFP^HI^ DN3 cells (119-fold increase) compared to WT mice (Fig. 3D). In contrast, the GFP^LO^ DN3 cell population in leukemic mice was not significantly expanded compared to WT mice (Fig. 3E).

Our scRNA-seq data indicated that dDN3 cells shared features of DN3a-stage thymocytes, while pDN3 cells resembled DN3b-stage thymocytes (Fig. 2G–I). To confirm whether the cell cycle–restricted GFP^HI^ DN3 leukemic cells resembled DN3a-stage thymocytes, we additionally co-stained pulse-chased *Tal1/Lmo2* leukemic mice for CD27, enabling us to distinguish between DN3a and DN3b cells [31]. This analysis revealed that approximately 67% of DN3 leukemic cells resided in the DN3a compartment, with the remaining 30% in the DN3b compartment (Fig. 3F, G). When gating on GFP^HI^ DN3 leukemic cells, 95% of all cell cycle–restricted DN3 cells were found to be DN3a (Fig. 3F, G). These findings align with our scRNA-seq gene expression data, indicating that dormancy is restricted to the leukemic DN3a population.

### Chemotolerance in *TAL1/LMO2* quiescent DN3 progenitors

**Fig. 4:**
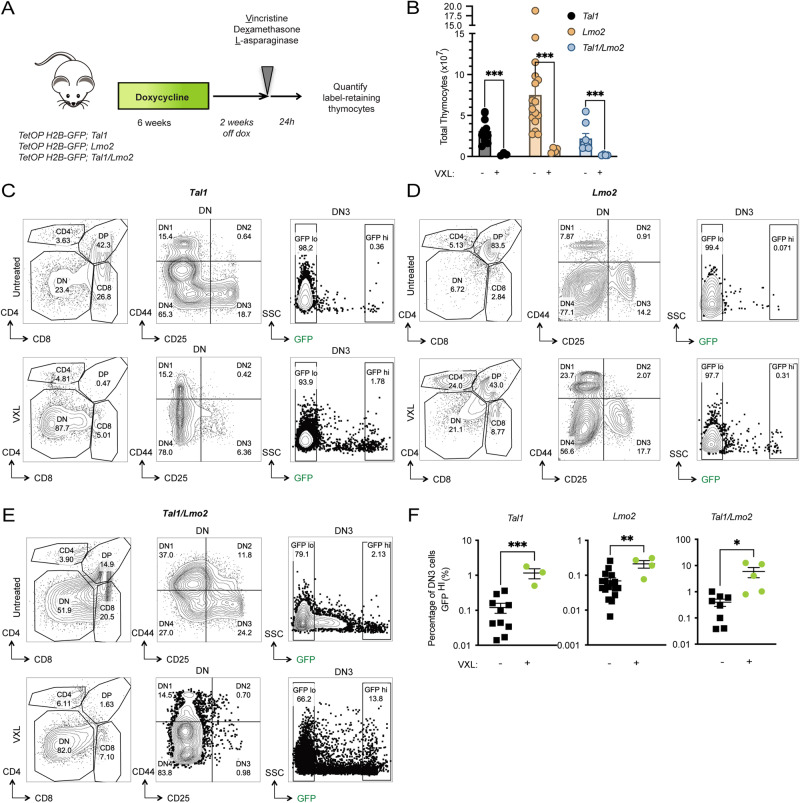
Dormant DN3 cells from preleukemic mice exhibit chemotolerance. **A** Schematic for testing chemotherapy sensitivity of preleukemic DN3 thymocyte subpopulations after pulse-chase. Mice were treated with vincristine (0.15 mg/kg), dexamethasone (5 mg/kg), and L-asparaginase (1000 U/kg) (VXL). **B** Thymocyte cellularity in untreated preleukemic mice and preleukemic mice treated with vincristine (0.15 mg/kg), dexamethasone (5 mg/kg), L-asparaginase (1000 U/kg) (VXL). **C** Representative flow cytometry plots of untreated (top row) or VXL-treated (bottom row) *Tal1*, **D** *Lmo2* untreated (top row) or VXL-treated (bottom row) or **E** *Tal1/Lmo2* untreated (top row) or VXL-treated (bottom row) mice after pulse-chase. Left column shows CD4/8 staining of preleukemic thymus. Middle column shows CD25 and CD44 staining of DN thymocytes. Right column shows GFP signal of DN3 cells after completion of pulse period. **F** Quantification of GFP^HI^ cells as a fraction of all DN3 thymocytes in untreated vs. VXL-treated *Tal1* (*n* = 3), *Lmo2* (*n* = 4), or *Tal1/Lmo2* (*n* = 5) thymi. Data represent mean $\pm \,$SEM. *p < 0.05, **p < 0.01, ***p < 0.001.

The detection of dormant L-ICs raised the possibility that this population may be chemoresistant and thus contribute to disease relapse. To explore this, we administered a combination of vincristine, dexamethasone, and L-asparaginase (VXL) [33] to pulse-chased preleukemic mice 24 h before sacrifice. We then examined label retention in the surviving DN3 cells (Fig. 4A). As expected, VXL treatment led to a more than 10-fold reduction in thymocyte numbers across all genotypes (Fig. 4B). Analysis of the remaining DN3 cells in VXL-treated *Tal1, Lmo2*, and *Tal1/Lmo2* mice revealed significant increases in the frequency of GFP^HI^ cells, with respective fold changes of 3-, 8-, and 15 (Fig. 4C–F), when compared to untreated mice of the same genotype. These results indicate that preleukemic GFP^HI^ dDN3 cells exhibit chemotolerance, contrasting with the GFP^LO^ pDN3 population.

**Fig. 5:**
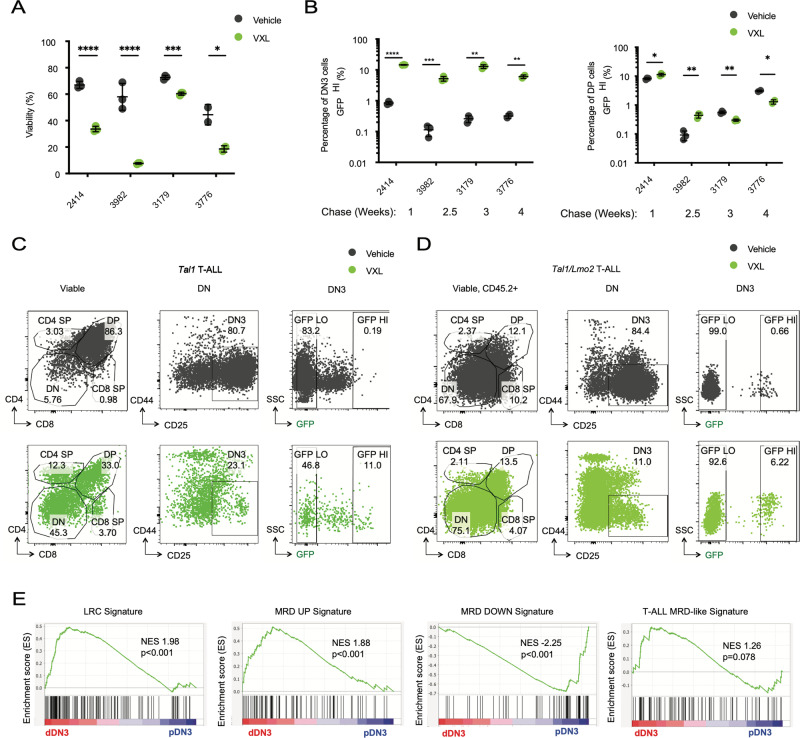
Dormant L-ICs are chemotolerant and enriched for ALL therapy resistance signatures. **A** Viability of pulse-chased *Tal1* (3982, 3179) and *Tal1/Lmo2* (2414, 3776) H2B-GFP leukemic cells treated ex vivo with vehicle (DMSO) or VXL (vincristine (5 ng/uL), dexamethasone (500 nM), and L-asparaginase (1 IU/mL) for 24 h **B** Quantification of GFP^HI^ cells in vehicle (gray) or VXL-treated (green) leukemic cultures as a percentage of all viable DN3 (left) or DP (right) cells. Representative flow cytometry plots for **C** *Tal1 H2B-GFP* (3179*)* and **D** *Tal1/Lmo2 H2B-GFP* (2414) T-ALL cells treated with vehicle (above) or VXL (below). Data represent mean $\pm \,$SD (*n* = 2-3 technical replicates). *p < 0.05, **p < 0.01, ***p < 0.001, ****p < .0001. **E** GSEA comparisons of dDN3 and pDN3 leukemic clusters to therapy resistant ALL signatures: LRC (label retaining cell), MRD (Minimal Residual Disease) UP, MRD DOWN (Ebinger et al. *Cancer Cell*, 2016), T-ALL MRD-like (Bortolozzi et al. *Br J Cancer*, 2018).

To assess whether dDN3 cells in leukemic mice also display chemotolerance, we treated pulse-chased H2B-GFP *Tal1* or *Tal1/Lmo2* leukemic cells with vehicle or VXL. This treatment resulted in significant reductions in leukemic cell viability and significant enrichment in GFP^HI^ DN3 cells compared to the vehicle control (Fig. 5A–D). Interestingly, VXL treatment had no significant effects on the percentage of GFP^HI^ double positive (DP) leukemic cells (Fig. 5B), despite our finding that Tal1 and Tal1/Lmo2 increased the percentage of GFP^HI^ DP cells in preleukemic mice (Supplementary Fig. 4). These data suggest that not all label retaining cells are chemotolerant and that chemotherapy leads to the specific enrichment of dormant DN3 leukemic cells.

To investigate the potential relevance of mouse dDN3 cells in the context of relapse, we compared our mouse dDN3 leukemic signature with published signatures associated with therapy resistance in ALL: ALL patient label-retaining cells [34], which have previously been demonstrated to mediate therapy resistance and relapse in a preclinical model, B-ALL cells obtained at MRD [34], and T-ALL MRD-like diagnostic samples [35]. Interestingly, dDN3 leukemic cells were enriched for all three therapy resistance signatures, suggesting that the population we identified through scRNA-seq and validated via nucleosome labeling may provide insights into treatment failure in T-ALL patients (Fig. 5E).

### T-ALL patient L-ICs are chemotolerant and exhibit cell cycle restriction

During normal human thymopoiesis, TCR-β rearrangement coincides with the upregulation of CD1a expression. This stage aligns with the DN3 stage in mice (Supplementary Fig. 5A) [36]. Previous studies on primary T-ALL patient samples indicated that CD7^+^CD1a^−^ population is enriched for L-IC activity, suggesting that T-lineage-committed DN3-like progenitors harbor L-ICs in T-ALL patients, mirroring findings in mouse models [6].

We examined primary and PDX T-ALL samples for the expression of L-IC markers. While other potential L-IC markers (e.g., CD34) [5] were not consistently detected, our analysis revealed the presence of the CD7^+^CD1a^−^ population across all diagnostic (mean 52 ± 13%) and relapsed/refractory (mean 49 ± 15%) samples examined. Notably, this population was also the predominant immunophenotype (85 ± 10%) among the early T-cell precursor -ALL subtype (Supplementary Fig. 5B).

**Fig. 6:**
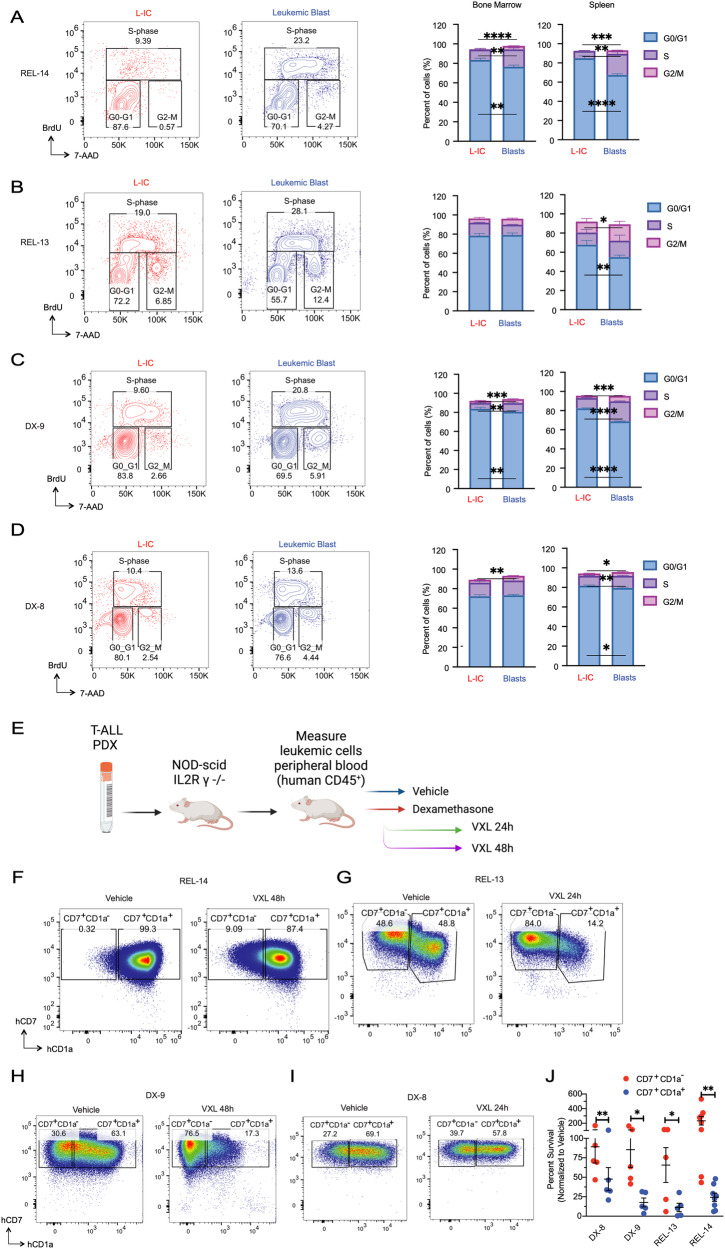
bHLH/LMO human L-ICs are quiescent and chemotolerant. (Left) Representative FACS plot showing decreased in vivo BrdU incorporation of L-ICs compared to leukemic blasts and (Right) quantification of BrdU/7-AAD cell cycle profile. Data are from REL-14 (**A**; *n* = 5), REL-13 (**B**; *n* = 3), DX-9 (**C**; *n* = 5), and DX-8 (**D**; *n* = 4). NSG mice engrafted with relapsed (REL) or diagnostic (DX) T-ALL PDX samples were pulsed with BrdU 1 h prior to sacrifice. Cell cycle profiles of human L-ICs (CD45^+^/CD1a^-^ red, left) or blasts (CD45^+^/CD1a^+^ blue, right) are shown. **E** Schematic for assessing chemotherapy response of human T-ALL subpopulations. **F** Representative FACS plots for REL-14 treated with vehicle (DMSO) or Dex (5 mg/kg)/VXL for 48 h, **G** REL-13 treated with vehicle (DMSO) or Dex/VXL for 24 h **H** DX-9 treated with vehicle (DMSO) or VXL for 24 h. **I** DX-8 treated with vehicle (DMSO) or VXL for 24 h. **J** Percent survival of human L-ICs (CD45^+^/CD1a^-^ red) vs blasts (CD45^+^/CD1a^+^ blue) are shown. *N* = 5, 5, 5, and 8 xenografts analyzed for DX-8, DX-9, REL-13 and REL-14, respectively. All comparisons by paired two-sided t-test. *p < 0.05, **p < 0.01, ***p < 0.001, ****p < .0001.

To determine whether hL-ICs, like their mouse counterparts, exhibit cell cycle restriction, we xenografted relapsed patient samples into NSG mice and utilized in vivo BrdU incorporation to quantitatively assess cell cycle dynamics. BrdU/7-AAD staining of the relapsed T-ALL sample (REL-14) demonstrated an increased proportion of G0/G1 cells and a corresponding decrease in S and G2/M phases cells among hL-ICs in comparison to the CD7^+^CD1a^+^ blasts (Fig. 6A). A similar trend was observed in an additional TAL/LMO relapsed sample (REL-13), wherein there was a notable increase in G0/G1 among the hL-ICs in the spleen (Fig. 6B), which was more predominant following VXL treatment (Supplementary Fig. 5C). These findings were subsequently confirmed in two additional T-ALL samples obtained at diagnosis (DX-9 and DX-8), indicating that quiescent hL-ICs are present in chemotherapy naïve patient samples (Fig. 6C, D). While the extent of differential cell cycle restriction showed inter-patient variability, the general observation was that L-ICs exhibited a higher degree of cell cycle restriction when compared to the leukemic blasts.

We then tested whether hL-ICs were differentially sensitive to chemotherapy. NSG mice were xenografted with relapsed and diagnostic T-ALL samples and, at a high leukemic burden, were randomized into treatment groups (Fig. 6E). Mice were treated with either vehicle or dexamethasone. To mimic standard ALL therapy, a separate group received dexamethasone followed by VXL chemotherapy, which significantly reduced leukemic burden compared to vehicle controls (Fig. 6E, Supplementary Fig. 5D) [33]. Notably, analysis of the remaining viable REL-14 leukemic cells following dexamethasone treatment showed a decrease in CD7^+^CD1a^+^ cells, yet no discernible effect on the CD7^+^CD1a^−^ hL-IC population, consistent with published work (Supplementary Fig. 5E) [6]. Upon addition of VXL, hL-IC were significantly enriched among REL-14 and REL-13 xenografted mice (Fig. 6F, G; Supplementary Fig. 5F).

Comparable results were obtained with diagnostic T-ALL samples, DX-9 and DX-8 (Fig. 6H, I). Importantly, hL-ICs demonstrated significantly increased survival after VXL treatment compared to the CD7^+^CD1a^+^ population (Fig. 6J). These data underscore that the dexamethasone/VXL treatment was significantly more effective at eliminating proliferating CD7^+^CD1a^+^ cells compared to the cell cycle–restricted CD7^+^CD1a^−^ hL-ICs in both diagnostic and relapsed/refractory patient samples (Fig. 6J).

**Fig. 7:**
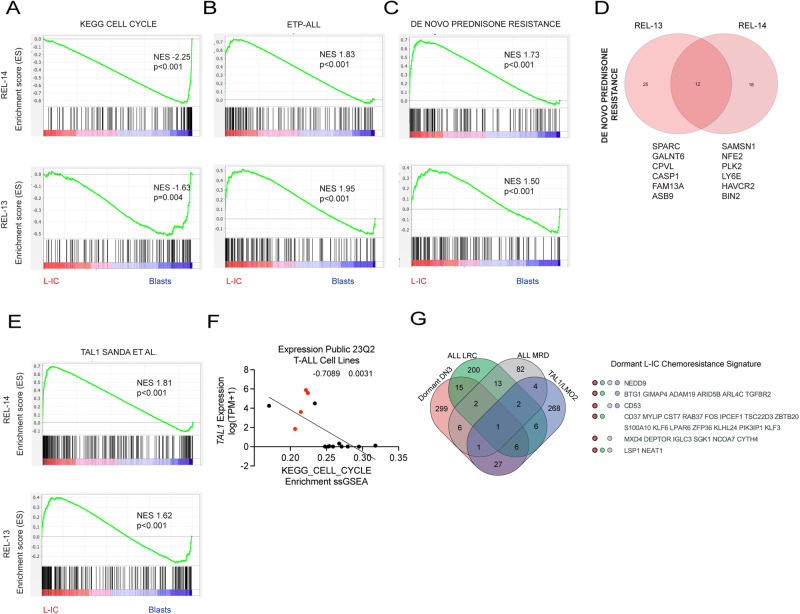
Relapsed bHLH/LMO human L-ICs are depleted for cell cycle genes and enriched for ETP-ALL, TAL1 and therapy resistance genes. **A** GSEA analysis showing depletion of KEGG cell cycle genes in hL-IC (CD7^+^CD1a^−^) compared to DP blasts (CD7^+^CD1a^+^) in REL-14 (above) and REL-13 (below). **B** GSEA analysis showing enrichment of of ETP-ALL genes (Zhang et al. *Nature*, 2012) in hL-IC (CD7^+^CD1a^−^) compared to DP blasts in REL-14 (above) and REL-13 (below). **C** GSEA analysis showing enrichment of de novo Prednisone Resistance genes (Paugh et al. *Nature Genetics*, 2015) in hL-IC compared to DP blasts in REL-14 (above) and REL-13 (below). **D** Overlap of shared leading edge de novo Prednisone Resistance Genes between REL-13 and REL-14. **E** GSEA analysis showing enrichment of TAL1 target genes (Sanda et al. *Cancer Cell*, 2012) in REL-14 (above) and REL-13 (below) hL-IC compared to DP blasts. **F** DepMap analysis showing *TAL1* expression is inversely correlated with KEGG cell cycle pathway scores in human T-ALL cells (*r* = −0.7089; *p* = 0.0031). Red circles indicate cell lines annotated as TAL1 positive. **G** Overlap of leukemic dDN3, MRD, and LRC (Ebinger et al. *Cancer Cell*, 2016), TAL1 (Sanda et al. *Cancer Cell*, 2012) and LMO2 (McCormack et al*. Science*, 2010) signatures.

To define the hL-IC signature in relapsed TAL/LMO T-ALL samples, we sorted CD7^+^CD1a^−^ hL-ICs from the CD7^+^CD1a^+^ DP leukemic cells from REL-14 and REL-13 patient xenografts (Supplementary Fig. 6A, B) and compared gene expression profiles (Supplementary Fig. 6C). As expected, *CD1A* expression was decreased in CD7^+^CD1a^−^ hL-ICs in both samples (Supplementary Fig. 6B). Concordant with BrdU incorporation data, DP cells were enriched for cell cycle genes compared to hL-ICs (Fig. 7A). In line with their premature developmental stage and observed therapy resistance, GSEA revealed enrichment of early T-cell precursor (ETP)-ALL and de novo prednisone resistance signatures in hL-ICs compared to DP blasts (Fig. 7B–D) [37]. hL-ICs were also enriched for TAL1 targets (Fig. 7E), suggesting that, as in the mouse model, TAL/LMO may promote hL-IC quiescence. Furthermore, expression of *TAL1* in human T-ALL cell lines was strongly anticorrelated with expression of cell cycle genes (Fig. 7F).

To hone-in on a consensus chemoresistance signature, we compared our leukemic dDN3 signature to published transcriptomes from primary ALL treatment resistant (LRC and MRD) samples and known TAL/LMO target genes. This analysis revealed several putative mediators of TAL1-induced quiescence and therapy resistance, including *NEDD9*, *BTG1, GIMAP4, ADAM19, ARID5B, ARL4C, TGFBR2*, and *CD53* (Fig. 7G). While *GIMAP4* and *ARID5B* have been validated as critical TAL1 targets [38, 39], the remaining genes identified by this approach have not been formally linked with TAL1 transformation. Interestingly, TGF-β was recently shown to promote the chemoresistance of dormant squamous cell carcinoma tumor-propagating cells [40], and *NEDD9* is implicated in TGF-β-induced growth inhibition [41]. *BTG1* is required for quiescence of naïve T-cells [42], thus it is possible that *BTG1* may also serve this role in chemotolerant L-ICs. Finally, *CD53* is overexpressed in *Lmo2* mouse leukemias and was shown to protect human TAL1+ JURKAT cells from apoptosis [43, 44]. Thus, this analysis converges on several *TAL1* targets that may mediate hL-IC quiescence and treatment resistance in T-ALL.

## Discussion

This study, conducted using T-ALL mouse models, expands on earlier findings that L-ICs are enriched in the DN3 population, but likely represent a rare subset [7, 8, 10, 45]. We profiled leukemic development in vivo using scRNA-seq and discovered a previously unrecognized dormant DN3 subpopulation that emerges during the preleukemic stage and persists after leukemic transformation. We then utilized in vivo nucleosome labeling to validate the existence of this quiescent DN3 population, revealing that *Tal1* and *Lmo2* cooperate to promote DN3 quiescence. During leukemic transformation, we observed a substantial expansion of GFP^HI^ (dormant) DN3 cells, while the population of GFP^LO^ (proliferative) DN3 cells remained relatively unchanged. These findings align with recent reports suggesting that ectopic Tal1 expression in thymic progenitors initially confers a growth disadvantage, which can only be overcome by additional mutations in the NOTCH1 or PI3K/AKT pathways [46, 47].

Regarding chemotherapy resistance and relapse, we demonstrate that dDN3 cells more readily survive ALL induction chemotherapy (VXL) compared to their actively proliferating counterparts. Furthermore, our comparative analysis of the dDN3 transcriptome with published signatures associated with therapy-resistant ALL cells obtained at MRD and those from ALL PDX samples characterized by label retention in NSG mice [34] revealed significant overlap, highlighting the potential translational relevance of our mouse L-IC signature to patient treatment failure. This analysis also allowed us to nominate TAL1 targets that may confer quiescence and chemotolerance, including multiple members of the TGF-β pathway (*TGFBR2, NEDD9*) and *BTG1*.

Our mouse L-IC results suggest that human thymic progenitors at a similar developmental stage may also function as L-ICs in T-ALL. Supporting this hypothesis is the presence of human CD7^+^CD1a^−^ progenitors, which, like their murine counterparts (dDN3 cells), represent the T-lineage committed stage before β-recombination [36] and have been associated with increased L-IC activity and dexamethasone resistance [6]. We consistently detected a CD7^+^CD1a^−^ hL-IC progenitor population among our T-ALL PDX samples (Supplementary Fig. 5B), further substantiating their potential involvement in L-IC activity. Treatment with dexamethasone followed by VXL lead to a robust enrichment of hL-ICs in both treatment naïve diagnostic and heavily pre-treated relapsed T-ALL PDX samples (Fig. 6).

Our cell cycle analysis revealed enrichment of G0/G1 cells and a scarcity of S- and G2/M-phase cells among hL-ICs, which was a variably observed phenotype in the diagnostic and relapsed T-ALL samples examined. Notably, cell cycle differences in the REL-13 sample were most predominant after VXL treatment, possibly indicating their potential to enter quiescence in response to cytotoxic therapy, distinguishing them from the CD7^+^CD1a^+^ blasts. Furthermore, the active cycling status of blasts following treatment suggests their susceptibility to iterative doses of therapy, which may, in turn, further enrich for the hL-IC population at the expense of leukemic blasts. Additionally, other L-IC features may contribute to chemotolerance, such as high expression of glucocorticoid resistance genes (Fig. 7). While *TAL1* target genes were enriched in hL-ICs, whether *TAL1* or other bHLH transcription factors directly promote quiescence in hL-IC deserves further inquiry.

### Supplementary information

Supplementary Figures and Methods

Supplementary Table 1

## Data Availability

Bulk and scRNA-seq are deposited in Gene Expression Omnibus GSE260697 and GSE260948, respectively and custom codes used to generate scRNA-seq data will be provided upon request.
