# Supplementary material for: The role of quiescent thymic progenitors in TAL/LMO2-induced T-ALL chemotolerance
Source: Leukemia. 2024 Mar 29;38(5):951–62. doi: 10.1038/s41375-024-02232-8 (PMC11073972; doi:10.1038/s41375-024-02232-8)
Supplement: Supplementary file 1 — Supplementary Figures and Methods [file 41375_2024_2232_MOESM1_ESM.pdf]

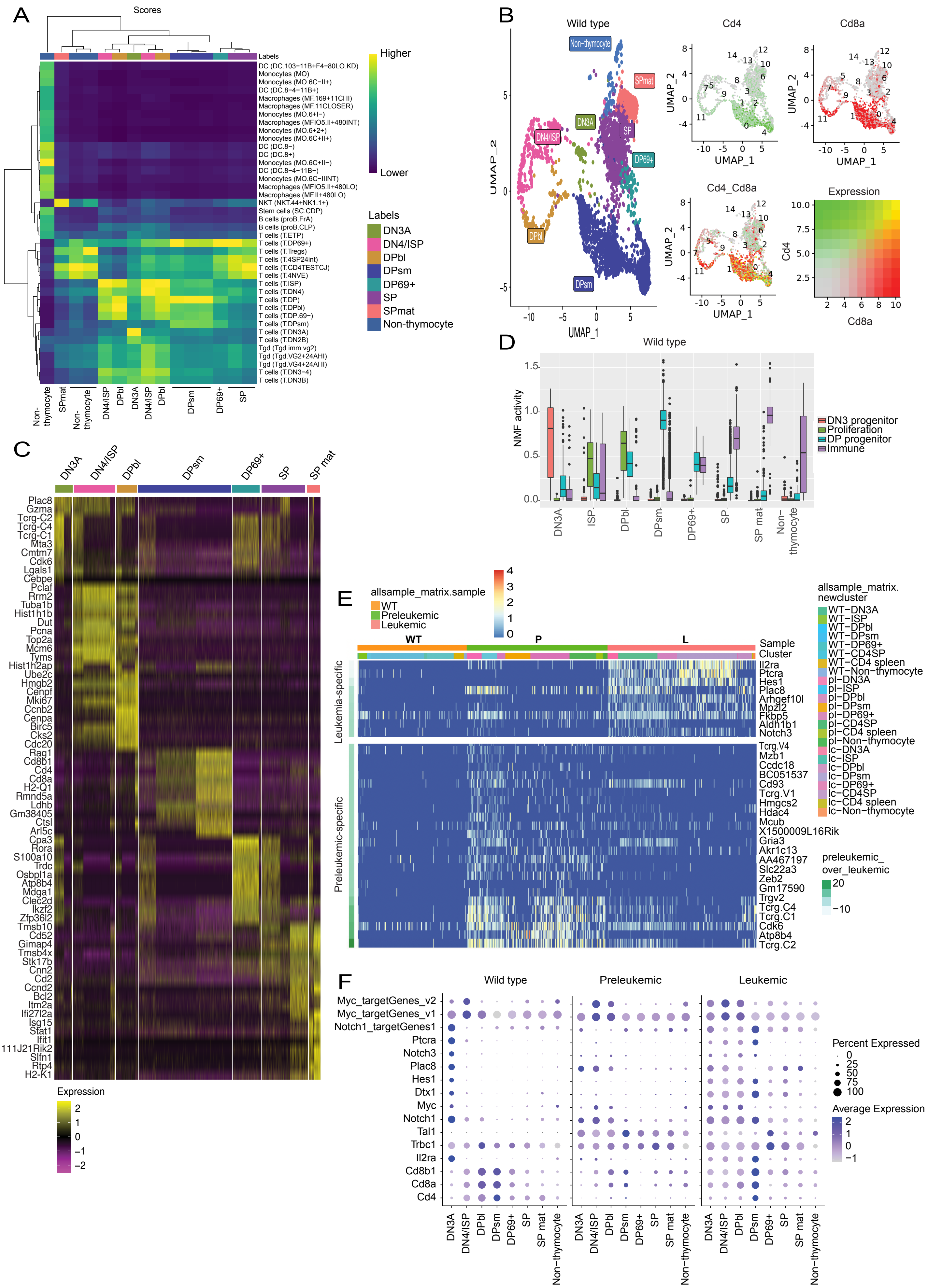
A
Scores
Labels
DC (DC.103−11B+F4−80LO.KD)
Monocytes (MO)
Monocytes (MO.6C−II+)
DC (DC.8−4−11B+)
Macrophages (MF.169+11CHI)
Macrophages (MF.11CLOSER)
Monocytes (MO.6+I−)
Macrophages (MFIO5.II+480INT)
Monocytes (MO.6+2+)
Monocytes (MO.6C+II+)
DC (DC.8−)
DC (DC.8+)
Monocytes (MO.6C+II−)
DC (DC.8−4−11B−)
Monocytes (MO.6C−IIINT)
Macrophages (MFIO5.II+480LO)
Macrophages (MF.II+480LO)
NKT (NKT.44+NK1.1+)
Stem cells (SC.CDP)
B cells (proB.FrA)
B cells (proB.CLP)
T cells (T.ETP)
T cells (T.DP69+)
T cells (T.Tregs)
T cells (T.4SP24int)
T cells (T.CD4TESTCJ)
T cells (T.4NVE)
T cells (T.ISP)
T cells (T.DN4)
T cells (T.DP)
T cells (T.DPbl)
T cells (T.DP.69−)
T cells (T.DPsm)
T cells (T.DN3A)
T cells (T.DN2B)
Tgd (Tgd.imm.vg2)
Tgd (Tgd.VG2+24AHI)
Tgd (Tgd.VG4+24AHI)
T cells (T.DN3−4)
T cells (T.DN3B)
Non-thymocyte
SPmat
Non-thymocyte
DN4/ISP
DPbl
DN3A
DN4/ISP
DPbl
DPsm
DP69+
SP
Higher
Lower
Labels
DN3A
DN4/ISP
DPbl
DPsm
DP69+
SP
SPmat
Non-thymocyte
B
Wild type
Non-thymocyte
SPmat
DN3A
SP
DN4/ISP
DP69+
DPbl
DPsm
UMAP_2
UMAP_1
Cd4
Cd8a
Cd4_Cd8a
Expression
Cd4
Cd8a
C
DN3A
DN4/ISP
DPbl
DPsm
DP69+
SP
SP mat
Plac8
Gzma
Tcrg-C2
Tcrg-C4
Tcrg-C1
Mta3
Cmtm7
Cdk6
Lgals1
Cebpe
Pclaf
Rrm2
Tuba1b
Hist1h1b
Dut
Pcna
Top2a
Mcm6
Tyms
Hist1h2ap
Ube2c
Hmgb2
Cenpf
Mki67
Ccnb2
Cenpa
Birc5
Cks2
Cdc20
Rag1
Cd8b1
Cd4
Cd8a
H2-Q1
Rmnd5a
Ldhb
Gm38405
Ctsl
Arl5c
Cpa3
Rora
S100a10
Trdc
Osbpl1a
Atp8b4
Mdga1
Clec2d
Ikzf2
Zfp36l2
Tmsb10
Cd52
Gimap4
Tmsb4x
Stk17b
Cnn2
Cd2
Ccnd2
Bcl2
Itm2a
Ifi27l2a
Isg15
Stat1
Ifit1
111J21Rik2
Slfn1
Rtp4
H2-K1
Expression
D
Wild type
NMF activity
DN3 progenitor
Proliferation
DP progenitor
Immune
DN3A
ISP
DPbl
DPsm
DP69+
SP
SP mat
Non−thymocyte
E
allsample_matrix.sample
WT
Preleukemic
Leukemic
WT
P
L
Sample
Cluster
Leukemia-specific
Preleukemic-specific
Il2ra
Ptcra
Hes1
Plac8
Arhgef10l
Mpzl2
Fkbp5
Aldh1b1
Notch3
Tcrg.V4
Mzb1
Ccdc18
BC051537
Cd93
Tcrg.V1
Hmgcs2
Hdac4
Mcub
X1500009L16Rik
Gria3
Akr1c13
AA467197
Slc22a3
Zeb2
Gm17590
Trgv2
Tcrg.C4
Tcrg.C1
Cdk6
Atp8b4
Tcrg.C2
allsample_matrix.
newcluster
WT−DN3A
WT−ISP
WT−DPbl
WT−DPsm
WT−DP69+
WT−CD4SP
WT−CD4 spleen
WT−Non−thymocyte
pl−DN3A
pl−ISP
pl−DPbl
pl−DPsm
pl−DP69+
pl−CD4SP
pl−CD4 spleen
pl−Non−thymocyte
lc−DN3A
lc−ISP
lc−DPbl
lc−DPsm
lc−DP69+
lc−CD4SP
lc−CD4 spleen
lc−Non−thymocyte
preleukemic_
over_leukemic
F
Wild type
Preleukemic
Leukemic
Myc_targetGenes_v2
Myc_targetGenes_v1
Notch1_targetGenes1
Ptcra
Notch3
Plac8
Hes1
Dtx1
Myc
Notch1
Tal1
Trbc1
Il2ra
Cd8b1
Cd8a
Cd4
DN3A
DN4/ISP
DPbl
DPsm
DP69+
SP
SP mat
Non−thymocyte
Percent Expressed
Average Expression

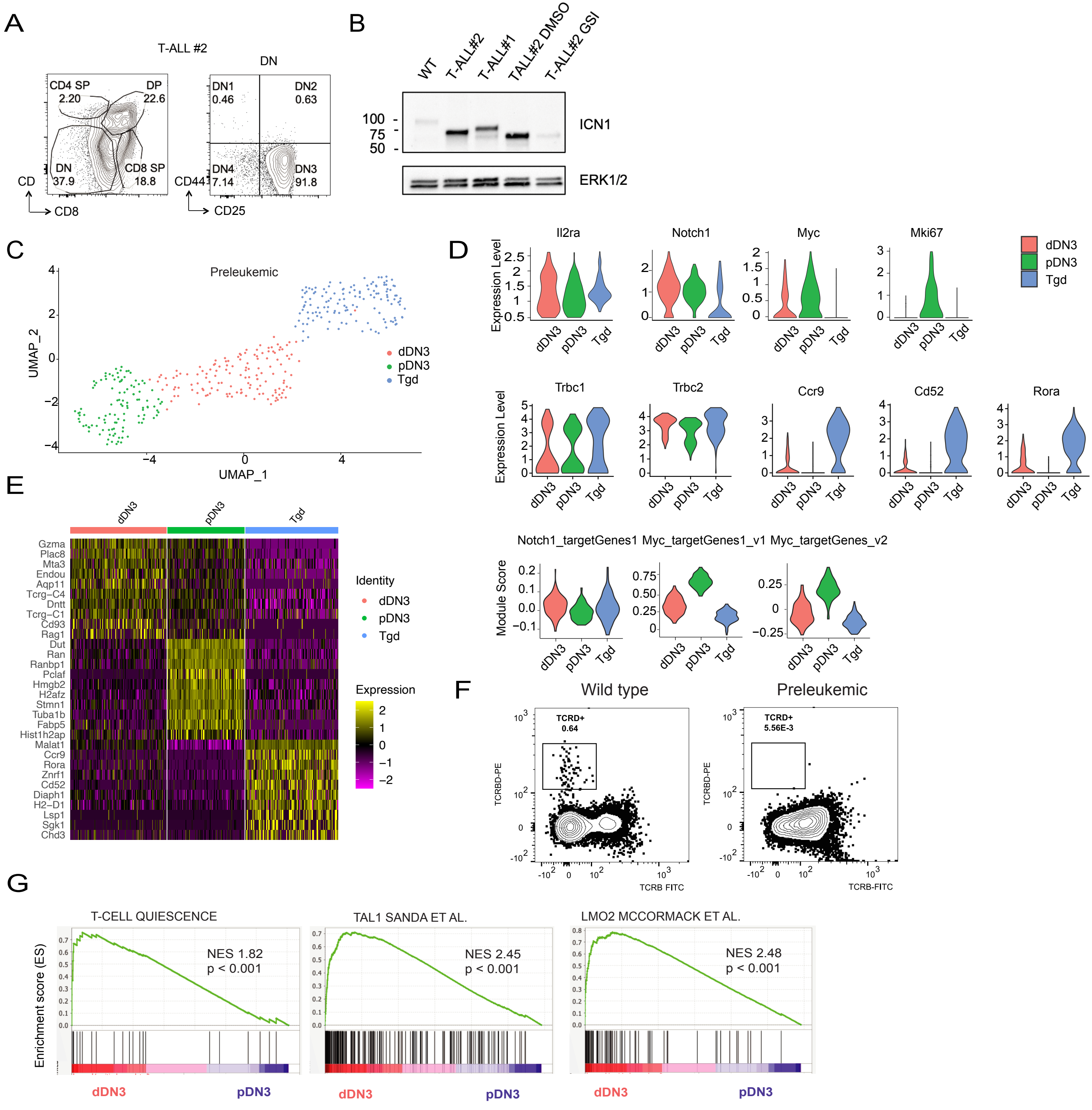

A
T-ALL #2
CD4 SP 2.20
DP 22.6
DN 37.9
CD8 SP 18.8
CD
CD8
DN
DN1 0.46
DN2 0.63
DN4 7.14
DN3 91.8
CD44
CD25
B
WT
T-ALL#2
T-ALL#1
TALL#2 DMSO
T-ALL#2 GSI
100
75
50
ICN1
ERK1/2
C
Preleukemic
UMAP_2
UMAP_1
dDN3
pDN3
Tgd
D
Il2ra
Notch1
Myc
Mki67
Expression Level
Trbc1
Trbc2
Ccr9
Cd52
Rora
Notch1_targetGenes1
Myc_targetGenes1_v1
Myc_targetGenes_v2
Module Score
E
Gzma
Plac8
Mta3
Endou
Aqp11
Tcrg−C4
Dntt
Tcrg−C1
Cd93
Rag1
Dut
Ran
Ranbp1
Pclaf
Hmgb2
H2afz
Stmn1
Tuba1b
Fabp5
Hist1h2ap
Malat1
Ccr9
Rora
Znrf1
Cd52
Diaph1
H2−D1
Lsp1
Sgk1
Chd3
Identity
Expression
F
Wild type
Preleukemic
TCRD+ 0.64
TCRD+ 5.56E-3
TCRBD-PE
TCRB FITC
TCRB-FITC
G
T-CELL QUIESCENCE
NES 1.82
p < 0.001
TAL1 SANDA ET AL.
NES 2.45
p < 0.001
LMO2 MCCORMACK ET AL.
NES 2.48
p < 0.001
Enrichment score (ES)
dDN3
pDN3

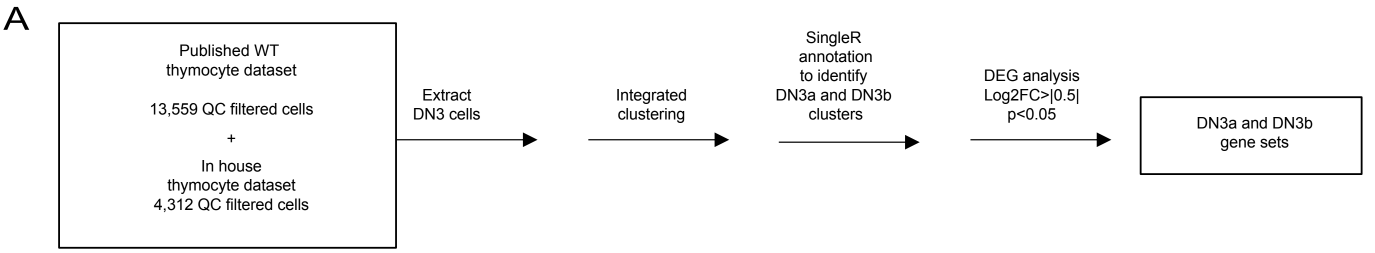

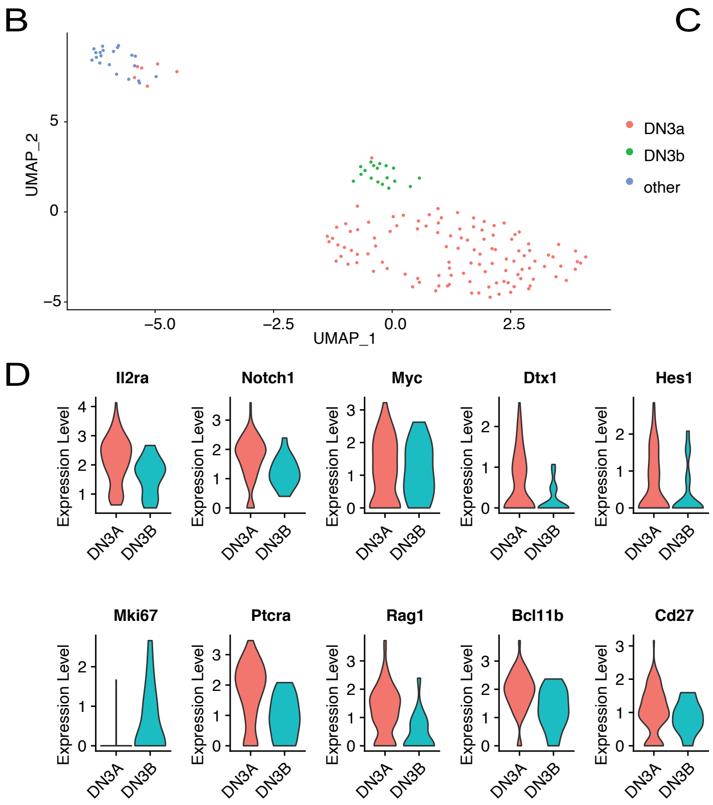

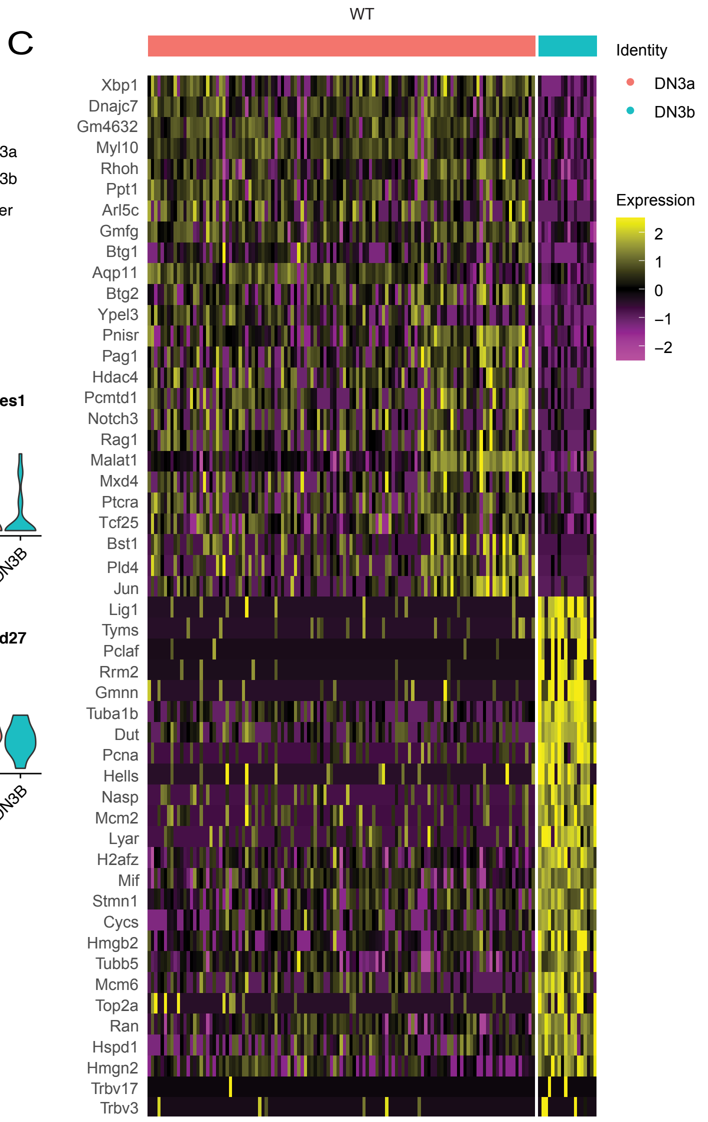

E

non-cell cycle genes

Comparison:	WT DN3a (NES)	WT DN3b (NES)
Dormant DN3 *Preleukemic*	2.90	−2.87
Dormant DN3 *Leukemic*	2.60	−3.00

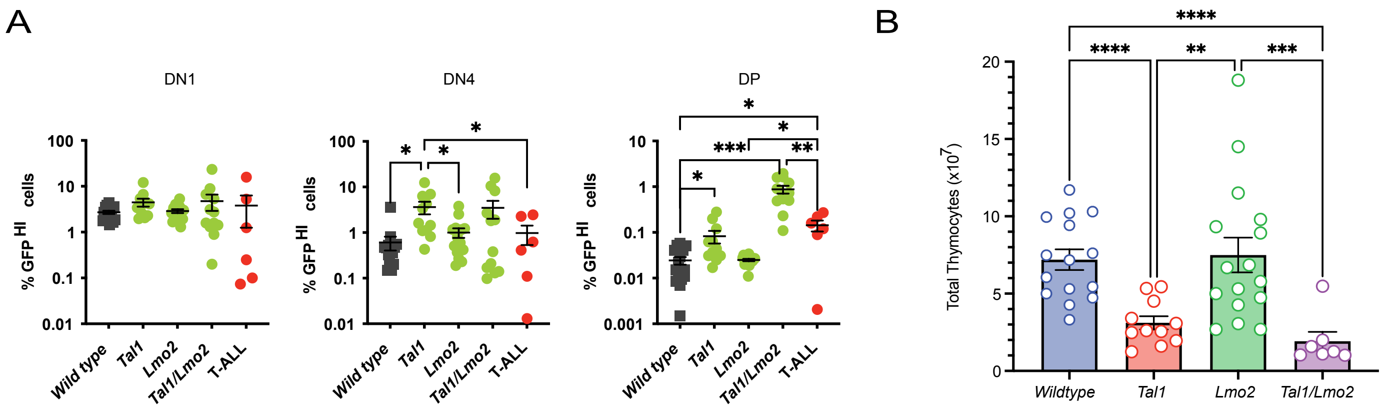

A
DN1
DN4
DP
100
10
1
0.1
0.01
0.001
% GFP HI cells
Wild type
Tal1
Lmo2
Tal1/Lmo2
T-ALL
*
**
***
B
Total Thymocytes (x10^7)
20
15
10
5
0
Wildtype
Tal1
Lmo2
Tal1/Lmo2
****
**
***

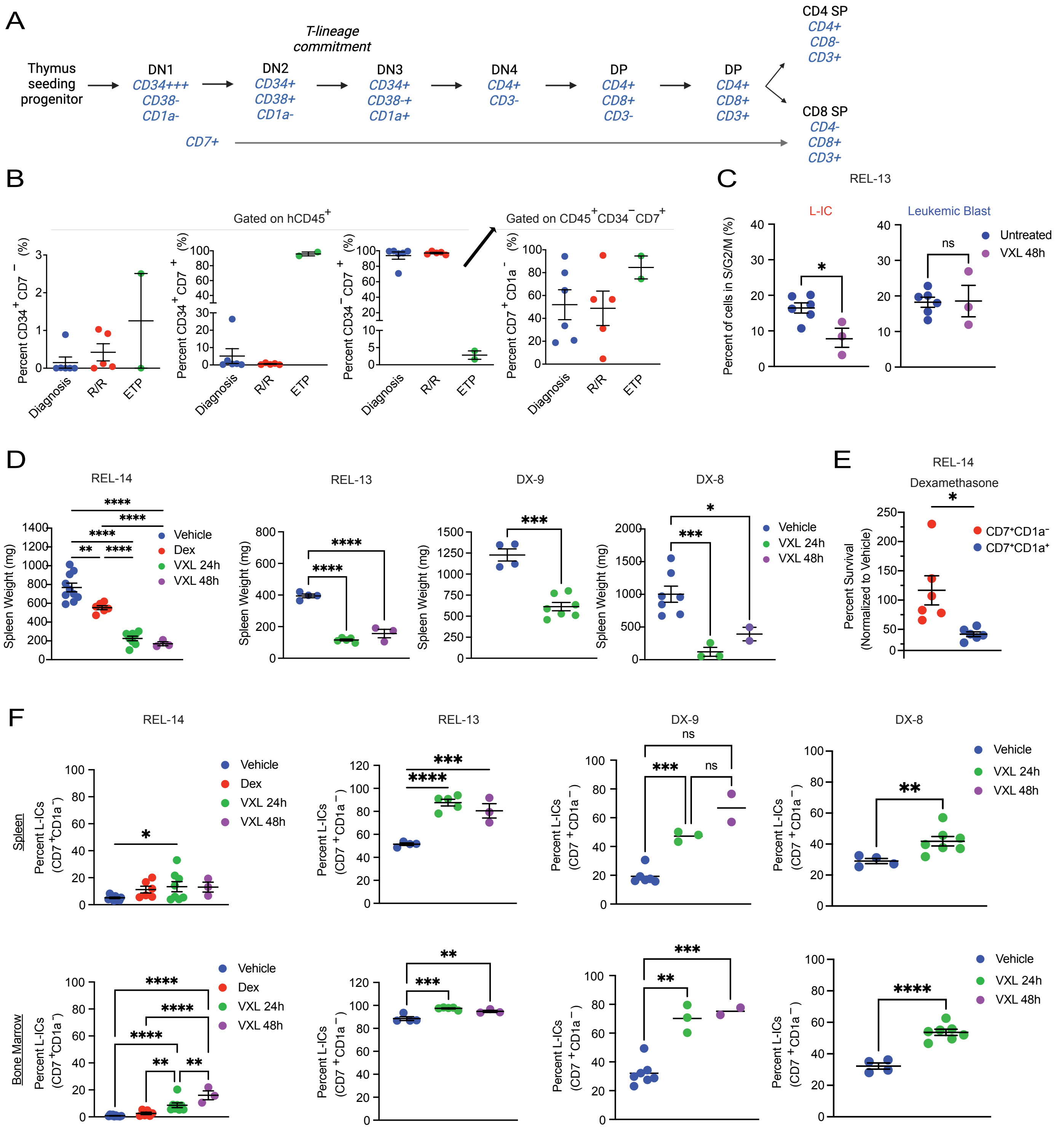
A
Thymus seeding progenitor
DN1 CD34+++ CD38- CD1a-
T-lineage commitment
DN2 CD34+ CD38+ CD1a-
DN3 CD34+ CD38-+ CD1a+
DN4 CD4+ CD3-
DP CD4+ CD8+ CD3-
DP CD4+ CD8+ CD3+
CD4 SP CD4+ CD8- CD3+
CD8 SP CD4- CD8+ CD3+
CD7+
B
Gated on hCD45+
Gated on CD45+CD34−CD7+
Percent CD34+CD7− (%)
Percent CD34+CD7+ (%)
Percent CD34−CD7+ (%)
Percent CD7+CD1a− (%)
Diagnosis
R/R
ETP
C
REL-13
L-IC
Leukemic Blast
Percent of cells in S/G2/M (%)
Untreated
VXL 48h
ns
D
REL-14
REL-13
DX-9
DX-8
Spleen Weight (mg)
Vehicle
Dex
VXL 24h
VXL 48h
E
REL-14
Dexamethasone
Percent Survival (Normalized to Vehicle)
CD7+CD1a−
CD7+CD1a+
F
REL-14
REL-13
DX-9
DX-8
Spleen
Bone Marrow
Percent L-ICs (CD7+CD1a−)
Vehicle
Dex
VXL 24h
VXL 48h
ns

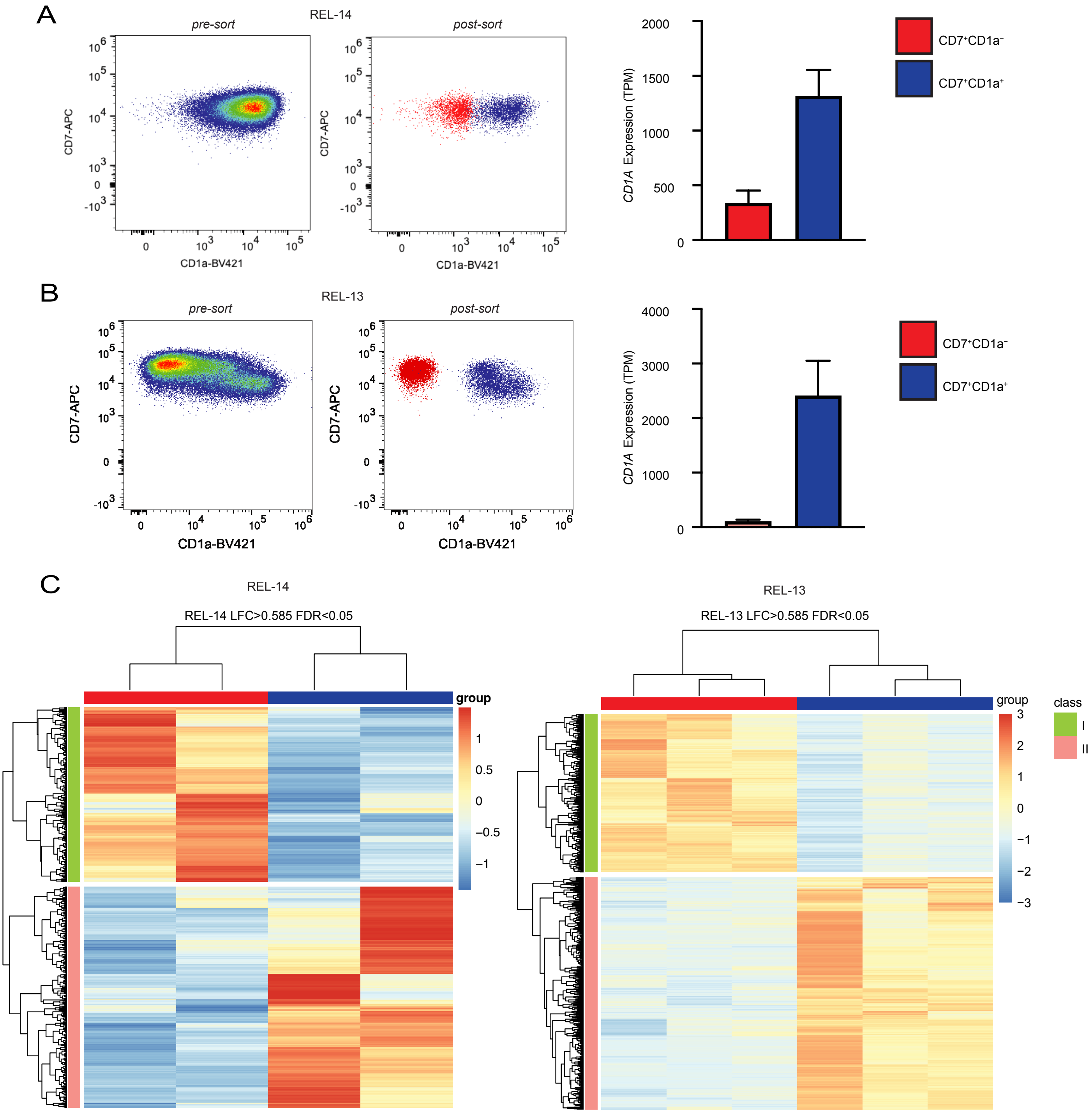

A
REL-14
pre-sort
post-sort
CD7-APC
CD1a-BV421
CD1A Expression (TPM)
CD7+CD1a-
CD7+CD1a+
B
REL-13
pre-sort
post-sort
CD7-APC
CD1a-BV421
CD1A Expression (TPM)
CD7+CD1a-
CD7+CD1a+
C
REL-14
REL-14 LFC>0.585 FDR<0.05
group
REL-13
REL-13 LFC>0.585 FDR<0.05
group
class
I
II

Supplementary Figure 1 scRNA-seq captures developmental perturbations associated with TAL1/LMO-mediated leukemogenesis. **A,** Hierarchical clustering of WT thymus using SingleR annotations. Color reflects confidence of SingleR annotations for individual clusters. Also see supplemental Table 1. **B,** UMAP of WT thymus showing cluster annotations (above) and feature plots for *Cd4* (green), *Cd8a* (red), or *Cd4/Cd8a* combined (yellow). Heatmap indicates extent of *Cd4/Cd8a* co-expression. **C,** Heatmap showing top 10 WT DEGs between clusters. **D,** cNMF analysis of wild type thymocytes identifies developmental stage-specific gene expression programs. Distribution of NMF activity for individual cells is plotted for each cluster. **E,** DN3 progenitor activity genes were ranked with respect to specificity to leukemic or preleukemic cells. Heatmap shows expression of top genes across integrated clusters for WT (left, orange), preleukemic (middle, green) or leukemic (right, salmon) cells. **F,** Dot plots showing expression of indicated genes or modules for WT, PL, and leukemic thymus.

Supplementary Figure 2. Identification of dormant and proliferative and g/d DN3 subpopulations in preleukemic thymus**. A,** Immunophenotype of T-ALL#2 (included in Figure 2 analysis) is shown. Second panel is gated on the leukemic CD4/8 double negative population. **B,** Immunoblot showing expression of truncated intracellular Notch1 (ICN1) in T-ALL samples using Val1744 antibody. T-ALL#2 leukemic cells were treated 48h with DMSO vehicle or 1uM GSI. Erk1/2 expression is shown as a loading control. **C**, UMAP plot of preleukemic (PL) DN3 thymocytes detects 3 clusters: dormant DN3 (dDN3), proliferative DN3 (pDN3), and DN3-gamma delta (Tgd). **D,** Top two panels, violin plots showing log2-transformed mRNA expression of indicated genes for PL DN3 clusters (above). Bottom panel, Notch1 and Myc (v1 and v2) module scores for respective clusters (below). **E,** Heatmap showing top 10 DEG for respective PL DN3 clusters. **F,** FACS plot showing surface expression of TCRδ or TCRβ in WT or preleukemic *Tal1/Lmo2* thymus. Gated on single cells. Representative of n=2 thymi from WT or *Tal1/Lmo2* preleukemic mice. **G,** GSEA using published data sets of T-cell quiescence (Eltanbouly *et al. Science*, 2020) and TAL1(Sanda *et al. Cancer Cell*, 2012) and LMO2 target genes (McCormack *et al. Science*, 2010) comparing dDN3 and pDN3 clusters.

Supplementary Figure 3. *Tal1/Lmo2* dormant DN3 cells resemble DN3a thymocytes enriched for quiescence and self-renewal genes. **A,** Workflow for generating WT DN3a and DN3b gene sets. **B,** Integrated UMAP plot of WT thymocytes showing DN3a, DN3b and “other” clusters. **C,** Heatmap showing top 25 WT DN3a and DN3b DEG. **D,** Violin plots showing log2-transformed mRNA expression of lineage genes for WT DN3a and DN3b clusters. **E,** GSEA analysis comparing pseudobulked preleukemic and leukemic dDN3 and pDN3 cells to WT DN3a and DN3b signatures after removing cell cycle related genes (Mingueneau *et al. Nature Immunology*, 2013).

Supplementary Figure 4. Tal1 and Lmo2 promote DN3 label retention during leukemogenesis. **A,** Frequency of GFP^{HI} cells in indicated thymocyte subsets after 2-week chase in WT (n=18) *Tal1* (n=11), *Lmo2* (n=16), *Tal1*/*Lmo2* (n=12) mice, or 2-3-week chase in mice with T-ALL (n=9). **B,** Total thymocyte cellularity of indicated genotypes from (B) at 3.5 months. Error bars represent mean$\pm$SEM. *p<0.05, **p<0.01, ***p<0.001, ****p<0.0001.

Supplementary Figure 5. bHLH/LMO human L-ICs are quiescent and chemotolerant. **A,** Stages of mouse and human thymocyte development. **B,** Immunophenotype of T-ALL patient-derived xenografts (PDXs), stratified by diagnosis (blue, n=6), relapsed/refractory (R/R, red, n=5), and early T-cell precursor ALL (ETP; green, n=2) samples. **C,** Comparison of Ki67/DAPI staining of untreated (blue; n=6) or VXL treated REL-13 cells 48h post-treatment (purple, n=3). hL-IC population shows decreased S/G2/M staining after treatment. **D,** Quantification of leukemic burden by spleen weight measurement in NSG mice engrafted with indicated PDX samples,

treated with Vehicle (blue), Dexamethasone 5 mg/kg (Red), Dexamethasone + VXL 24h (Green), or Dexamethasone + VXL 48h (purple). Vehicle n=10, 4, 4, 7, Dexamethasone n=6, Dexamethasone+VXL 24h n=8, 5, 7, 3 and Dexamethasone+VXL 48h n=3, 3, 2, 2 **E**, Percentage of REL-14 $CD7^{+}CD1a^{-}$ (hL-IC; red) vs. $CD7^{+}CD1a^{+}$ (blue) surviving dexamethasone therapy compared to xenografted mice treated with vehicle. **F,** Percentage of all viable leukemic cells hL-ICs in spleen (above row) or bone marrow (bottom row), after the indicated treatment. Data from 2 or 3 independent cohorts of NSG mice are shown for each PDX sample. One-way ANOVA was used to compare leukemic burden in (D) and enrichment of hL-IC with chemotherapy shown in (F). Paired two-tailed t-test was used to compare survival after VXL treatment between leukemic subpopulations (E). All error bars represent mean±SEM. *p<0.05, **p<0.01, ***p<0.001, ****p<0.0001.

Supplementary Figure 6. Relapsed bHLH/LMO human L-ICs are depleted for cell cycle genes and enriched for ETP-ALL, TAL1 and therapy resistance genes. **A,** Representative pre-sort (left) and post-sort (middle) immunophenotype profiles of CD7+CD1a- hL-IC (red) and CD7+CD1a+ DP blasts (blue) from REL-14 xenografts. Right, *CD1A* mRNA expression of respective populations (n=2 independent xenografts). **B,** Representative pre-sort (left) and post-sort (middle) immunophenotype profiles of CD7+CD1a- hL-IC (red) and CD7+CD1a+ DP blasts (blue) from REL-13 xenografts. Right, *CD1A* mRNA expression of respective populations (n=3 independent xenografts). **C,** Unsupervised hierarchical clustering of sorted RNA-seq samples from REL-14 (left) and REL-13 (right) using L2FC 0.585 (Fold change of 1.5) and FDR<0.05 as filtering criteria. CD7+CD1a- hL-IC (red) and CD7+CD1a+ DP blasts (blue) samples cluster together. All error bars represent mean±SEM.

scRNA-seq analysis. In total, 5879 WT, 6964 preleukemic, 7726 T-ALL#1 leukemic, and 7737 T-ALL#2 leukemic cells were processed. Quality control involved three criteria for cell exclusion: (1) mitochondrial gene percentages ≥ 10%, (2) cells with ≤ 500 nGene, and (3) cells with nGene values ≥ mean + three times the standard deviation (SD) (to exclude doublets). Additionally, genes expressed in fewer than five cells were removed. Following filtering, the analysis encompassed 4312 WT, 5532 preleukemic, 5779 T-ALL#1 leukemic, and 2026 T-ALL#2 leukemic cells for further investigation. Post-QC WT, preleukemic, and leukemic T-ALL#1 cells were processed in aggregate and subsequent integrated UMAP clustering of cells was performed using Seurat, using 15 principle components (PC) with a resolution of 0.8, which revealed 15 transcriptionally distinct clusters. Differentially expressed genes (DEGs) from WT thymocytes (between clusters) were then used as a reference to annotate each cluster with SingleR, which correlates DEGs with reference Immgen gene expression data on sorted immune cell subsets to predict cluster annotations (supplemental Table 1)[1]. Three non-thymocyte clusters (Natural Killer T-cell (NKT), regulatory T cells (Treg), and Monocytes) and redundant clusters (i.e. annotated as the same cell type) were combined. Two CD4 single positive (SP) clusters were detected, thymic CD4 SP and spleen CD4 SP, likely representing two stages of CD4 SP cell maturity. Further inspection of these clusters revealed both cells that expressed *Cd4* but not *Cd8a*, and cells that expressed *Cd8a* but not *Cd4,* and we thus termed these clusters SP and "SP mature" (SP mat), respectively (supplemental Figure 1B). For clustering WT, preleukemic, and leukemic DN3 cells, cells that met the filtering parameters Cd4=0, Cd8a=0, CD44=0, Il2ra>0.5 were extracted, reclustered with Seurat, and projected on UMAP plots. "Pseudobulking" of WT DN3a and DN3b clusters, as well as preleukemic and leukemic dormant and proliferative DN3 clusters, was done using the edgeR package[2].

Bulk RNA-sequencing (RNA-seq) of human PDX subpopulations. Low passage relapsed REL-13 or REL-14 PDX samples were xenotransplanted into NOD scid gamma (NSG) mice. Following successful engraftment of leukemic cells, mice were euthanized and spleens were harvested, ACK lysed, washed in PBS, then stained in a 2% FBS / PBS solution with CD45, CD7, and CD1a antibodies, prior to sorting of CD7+ CD1a- and CD7+ CD1a- subpopulations by FACS. Respective samples were subsequently processed using the Qiagen (Germantown, MD) RNAeasy mini kit to extract RNA. RNA samples were further processed by Novogene Corporation (Sacramento, CA) using SMARTer RNA amplification kit and ultra-low input mRNA non-directional library preparation, prior to sequencing 15G per sample on the Novoseq 6000 platform. RNA-seq analysis was performed using OneStopRNAseq[3]. Specifically, FastQC and MultiQC[4] were used for raw reads quality control and QoRTs[5] for post-alignment quality control. Reads were aligned to the reference genome assembly, hg38, with star_2.7.5a[6] and annotated with gencode.v34.primary_assembly[7]. Aligned exon reads were counted toward gene expression with featureCounts_2.0.0[8] with default settings. Differential expression (DE) analysis was performed with DESeq2_1.28.1[9]. Within DE analysis, 'ashr' was used to create log2 Fold Change (LFC) shrinkage for each comparison[10]. Significantly differentially expressed genes (DEGs) were filtered with the criteria FDR < 0.05 and absolute log2 fold change (|LFC|) > 1.0. Heatmaps were created with pheatmap.

Consensus non-negative matrix factorization (cNMF). To identify gene expression programs in WT thymus, cNMFv1.34 was performed on log2-transformed SCTransformed counts with pseudocount of 1 generated by Seurat with python 3.7.10 (Cite SCTransform). The following parameters were used for cNMF: num_iter=20, num_highvar_genes=2000, seed=14. The stability and error plot was assessed to select k of 6. Density threshold of 2.00 and k = 6 was used to generate 6 gene activity programs. For plotting cNMF activity in Supplemental Figure 1D, usage matrix was used. For each of these gene expression program, top 100 genes based on z-scores were selected and plotted as a heatmap per integrated cluster. Each gene expression program

was annotated using HOMER Gene Ontology analysis and manual inspection. Four out of six programs were chosen as the other two showed enrichment of non-thymocyte genes. The top 100 genes from the annotated and selected gene expression programs were plotted also for preleukemic and leukemic thymi to examine dysregulated transcriptional changes related to WT thymocyte differentiation.

UMAP clustering of leukemic DN3 cells. Leukemic DN3 cells from two independent *Tal1/Lmo2* mouse T-ALLs were normalized by SCTransform and integrated using 3000 features using Seurat. DN3 cells were subsetted by using the filtering criteria *Cd4*=0, *Cd8a*=0, *Cd44*=0, *Il2ra*>0.5. 30 PCA dimensions were used to run UMAP and to find clusters with a resolution of 1. Resulting four clusters were merged into two based on manual inspection of *Mki67* expression.

Differential gene expression analysis. To perform differential gene expression analysis on integrated leukemic DN3 cells, pseudobulking was performed to sum up all the counts across cells within each replicate[11]. All genes without any reads were filtered out. Normalization factor was calculated using calcNormFactors and estimateDispersion was performed with robust set to TRUE. glmQLF was used for testing and Benjamini-Hochberg method was used to correct for multiple hypothesis testing. To plot the heatmap for Figure 2C**,** top 30 upregulated and downregulated DE genes determined by the product of logFC and log10 of FDR.

Leukemic and preleukemic specificity scores. For Supplemental Figure 1E, to quantify the specificity of expression of genes from the DN3 progenitor program, difference of percentages of cells that express each gene between preleukemic and leukemic thymi was calculated, such that positive values indicate specific expression to preleukemic thymus and the negative values indicate specific expression to leukemic thymus.

Generation of single cell DN3a and DN3b signatures. Unsupervised clustering of 17,871 QC-filtered WT DN3 cells (in-house data set and [12]) revealed three clusters, which were annotated using SingleR[1] (supplemental Figure 3A-B). Cluster 1, which was transcriptionally distinct compared to the other two clusters, was annotated as a mixture of gamma delta T-cells and Tregs, and thus excluded from further analysis. The majority of the remaining cells were observed in the cluster annotated as DN3a (87%), compared to the DN3b cluster (13%) (supplemental Figure 3B), consistent with published data[13] and our flow cytometry analysis of WT DN3a (FSC-A^{LO}, $CD27^{LO}$) and DN3b (FSC-A^{HI}, $CD27^{HI}$) populations (Figure 3B-C). Analysis of top DEGs revealed expected gene expression differences, with higher expression of *Notch3*, *Rag1*, and *Ptca* in DN3a cells and higher expression of proliferation-associated genes, including *Plcaf*, *Pcna*, and *Top2a* in the DN3b cluster (supplemental Figure 3D). To generate reference gene sets, genes with L2FC>|0.5| and FDR<0.05 were included, leading to 278 and 496 DEG for DN3a and DN3b, respectively.

H2B-GFP label retention studies. $\geq 2x10^7$ cells per thymus (approximate total thymus cellularity for *Tal1/Lmo2* mice) were stained and $\geq 10^7$ thymocyte singlets were analyzed per sample (coefficient of variation (CV) for detecting rare populations <5% for *Tal1* and *Tal1/Lmo2* DN3 thymocytes). $GFP^{HI/LO}$ gates were established using pulse control mice and applied to pulse-chased samples to determine percent of cells retaining H2B-GFP label. Absolute numbers were calculated by multiplying total live (trypan blue negative) cells x immunophenotype fraction x percentage of respective $GFP^{HI/LO}$ populations. Control mice were routinely sacrificed at the end of the pulse period to assess proper labeling with H2B-GFP. Label retention of DN1 thymocytes was used as an additional internal control for labeling and mice with <1% GFP^{HI} DN1 cells censored from the study. For experiment presented in Figure 4 (2-week chase), 1/18 *WT*, 0/11 *Tal1*, 1/16 *Lmo2*, and 2/14 *Tal1/Lmo2* mice were censored using these criteria.

Defining preleukemic and leukemic H2B-GFP mice. Kaplan-Meier survival curves were previously determined for *Tal1* and *Tal1/Lmo2* transgenic mouse strains[14,15]. Thymus cellularity, spleen weights, and gross appearance of bone marrow were documented for all WT and preleukemic mice at the time of sacrifice. Animals were classified as leukemic if they exhibited thymic expansion (cellularity > 1.5×10^8) and/or splenomegaly/infiltration of bone marrow with Thy1.2+ cells (by FACS) at the time of sacrifice. Leukemia was confirmed by transplanting 10^5 cells from the thymus into the tail vein of sublethally irradiated (650 cGy) congenic CD45.1+ (B6.SJL-*Ptprc*[a] *Pepc*[b]/BoyJ) recipients and determining that recipient hosts succumbed to leukemia with short latency.

GSEA analysis. Preranked GSEA analysis was performed using the GSEA (v4_3.0) desktop application with 1000 permutations. Rank files were prepared by ranking all detected genes according to L2FC. Hallmark gene sets were obtained from MSigDB: mh.all.v2023.1.Mm.symbols.gmt (mouse) and h.all.v2023.1.Hs.symbols.gmt (human)[16]. Mouse .rnk files were compared to human MSigDB gene sets by collapsing genes onto the Mouse Gene Symbol Remapping Human Orthologs MSigDB.v2023.1.Hs.chip. Curated gene sets are provided in supplemental Table 1. For comparison of mouse and human gene expression signatures, mouse genes were converted to human homologs using the SynGO gene conversion tool[17]. Mouse Genome Informatics (The Jackson Laboratory) was used for converting human gene sets into mouse homologs.

Statistical modeling of label retention data. We used R for the statistical analysis of DN3 GFP proportion and count (absolute cell number) data. Proportion of GFP positive cells was first logit transformed to homogenize the variance. Levene's test indicates that the assumption of homogeneity of variances was met. One-way analysis of variance (ANOVA) with Completely Randomized Design shows that there is significant difference among groups (F-test p = 6.436e-06). A set of pre-defined contrasts were performed under the ANOVA framework using lsmeans package[18]. To adjust for multiple comparisons, p-values were adjusted using the Benjamini-Hochberg (BH) method[19]. Number of cells was modeled as negative binomial distribution using generalized linear model.

Relabeling leukemic H2B-GFP cells in vivo. To re-establish H2B-GFP expression in leukemic mice, congenic mice were transplanted with of $0.5\text{-}1 \times 10^5$ cells and provided doxycycline in the drinking water. As above, pulse control mice were sacrificed at intervals between 3-4 weeks, prior to withdrawal of doxycycline, using CD45.2 as a marker of leukemic cells to assess labeling efficiency. Leukemic cells were harvested from the bone marrow and label retention was assessed by sacrificing mice 1 week later.

Ex vivo chemotherapy treatment. Leukemic cells from pulse-chased *Tal1* or *Tal1/Lmo2* mice were cultured in RPMI 1640 (Gibco) supplemented with 20% FBS (Cytiva HyClone), 1% Penicillin-streptomycin (Gibco), IL-2 (10 ng/mL), IL-7 (10 ng/mL), SCF (2 ng/mL), and FLT3L (5 ng/mL) (Peprotech). Duplicate or triplicate cultures of $4\text{-}10 \times 10^6$ cells were treated with DMSO or vincristine (5 ng/uL), dexamethasone (500 nM), and L-asparaginase (1 IU/mL) for 24 hours. Cells were harvested and stained with Ghost Dye Violet 510 (Tonbo Bioscience) according to manufacturer's instructions, as well as CD4/8/25/44 antibodies (see below), and then all cells from each replicate were analyzed by flow cytometry. Survival was calculated by dividing the absolute number of viable (Ghost 510 neg) GFP^{HI} or GFP^{LO} DN3 leukemic cells counted by flow cytometry in VXL-treated cultures by the mean of the absolute number of cells counted in DMSO cultures, then multiplying by 100%. In the case of #2414 and #3776, pulse-chased cells were obtained from the BM of leukemic mice and thus CD45.2 (for #2414) or Thy1.2 (#3776) were included in the immunostaining panel as a lineage marker. To obtain pulse-chased #2414 leukemic cells, 10^5 primary leukemic cells from the thymus were xenotransplanted into congenic

(CD45.1+) mice via tail vein, pulsed with doxycycline for 4 weeks, followed by a one-week chase period.

In vivo chemotherapy treatment. Vincristine sulfate (0.15 mg/kg), dexamethasone (5 mg/kg), and L-asparaginase (1000 U/kg) (Sigma, VXL) were dosed via intraperitoneal injection as previously described[20]. For PDX studies, at high leukemic burden (see supplemental Figure 5B), mice were randomized to receive vehicle (17% DMSO in PBS), 5 mg/kg dexamethasone, or 5 mg/kg dexamethasone followed by one dose of VXL chemotherapy. Mice were euthanized 24-48 hours after treatment and bone marrow (BM) and spleen were harvested, lysed with ACK buffer, and stained for cell surface markers.

FACS analysis. The following antibodies were used to stain mouse thymocytes and leukemic cells: BioLegend CD45.2-BV421 (1:20, Clone 104), CD90.2-BV421 (1:20, 53-2.1), CD4-PerCP-Cy5.5 (1:200, RM4-5), CD8-APC-Cy7 (1:200, 53-6.7), CD27-PE (1:200, LG.3A10), TCRβ-FITC (1:200, H57-597), TCRγ/δ (GL3, 1:200); BD Pharmingen CD8-FITC (1:200, 53-6.7), CD25-PE-Cy7 (1:200, PC61), CD44-APC (1:200, IM7). The following antibodies were used to stain human PDX cells: BioLegend CD45-BV510 and -PE-Cy7 (1:20 and 1:40, respectively, 2D1), CD7-APC (1:100, CD7-B67), CD1a-BV421 and -APC (1:400 and 1:20, respectively, HI149), and KI67-PE (1:40 #350504). 7-AAD was purchased from eBioscience. FACS analysis was performed using BD LSR II and BD Celesta flow cytometers. Cell sorting was performed on SONY SH800 and BD FACS Aria II. Fcs files were exported and analyzed using FlowJo v10 (BD).

Immunoblotting. Whole cell protein lysates were prepared by lysing cells in RIPA buffer containing Roche complete protease inhibitor (Millipore Sigma), sodium fluoride and sodium orthovanadate. Lysates were run on 4-12% Bis Tris polyacrylamide gels (Novex), transferred onto PVDF membranes, blocked in 5% milk, and incubated overnight at 4°C with the following antibodies: Cleaved Notch1 (V1744) (Cell Signaling, #4147S, 1:1,000) and β-Actin (Cell Signaling #3700 1:1,000). Membranes were subsequently incubated at room temperature 30' with appropriate rabbit and mouse HRP-conjugated secondary antibodies (GE Healthcare, 1:5,000).For nucleosome label retention studies, 6-week-old wild type (WT) C57B6/J, *Tal1, Lmo2* or *Tal1/Lmo2*; *H2B-GFP*$^{KI/+}$, *rTA*$^{KI/+}$mice (Jackson Laboratory; #016836) were pulsed for 6 weeks with doxycycline (2 g/L Sigma, D9891) to induce expression of H2B-GFP, followed by a 2-week chase to identify GFP^{HI} (dormant) and GFP^{LO} (proliferative) cells.

Mouse studies. Both male and female FVB/N and C57B6/J mice were used in this study. Female NSG mice were used for engraftment of PDX samples. For H2B-GFP label retention studies, an estimate of 0.1% GFP HI DN3 cells was used, with a standard deviation of 1.0 and an expected mean of 1.0% among transgenic mice. Using a power of 0.8 and significance threshold of 0.05, a sample size of approximately 10 control (WT) mice and 10 transgenic mice was chosen in order to detect this expected difference.

1. Aran, D. *et al.* Reference-based analysis of lung single-cell sequencing reveals a transitional profibrotic macrophage. *Nat Immunol* **20**, 163–172 (2019).

2. Robinson, M. D., McCarthy, D. J. & Smyth, G. K. edgeR: A Bioconductor package for differential expression analysis of digital gene expression data. *Bioinformatics* **26**, 139–40 (2009).

3. Li, R., Hu, K., Liu, H., Green, M. R. & Zhu, L. J. OneStopRNAseq: A Web Application for Comprehensive and Efficient Analyses of RNA-Seq Data. *Genes (Basel)* **11**, 1–14 (2020).

4. Ewels, P., Magnusson, M., Lundin, S. & Käller, M. MultiQC: summarize analysis results for multiple tools and samples in a single report. *Bioinformatics* **32**, 3047–3048 (2016).

5. Hartley, S. W. & Mullikin, J. C. QoRTs: a comprehensive toolset for quality control and data processing of RNA-Seq experiments. *BMC Bioinformatics* **16**, (2015).

6. Dobin, A. *et al.* STAR: ultrafast universal RNA-seq aligner. *Bioinformatics* **29**, 15–21 (2013).

7. Harrow, J. *et al.* GENCODE: the reference human genome annotation for The ENCODE Project. *Genome Res* **22**, 1760–1774 (2012).

8. Liao, Y., Smyth, G. K. & Shi, W. featureCounts: an efficient general purpose program for assigning sequence reads to genomic features. *Bioinformatics* **30**, 923–930 (2014).

9. Love, M. I., Huber, W. & Anders, S. Moderated estimation of fold change and dispersion for RNA-seq data with DESeq2. *Genome Biol* **15**, (2014).

10. Stephens, M. False discovery rates: a new deal. *Biostatistics* **18**, 275–294 (2017).

11. Squair, J. W. *et al.* Confronting false discoveries in single-cell differential expression. *Nat Commun* **12**, (2021).

12. Ramstead, A. G. *et al.* Mitochondrial Pyruvate Carrier 1 Promotes Peripheral T Cell Homeostasis through Metabolic Regulation of Thymic Development. *Cell Rep* **3;30**, 2889-2899.e6 (2020).

13. Taghon, T., Yui, M. A., Pant, R., Diamond, R. A. & Rothenberg, E. V. Developmental and molecular characterization of emerging β- and γδ-selected pre-T cells in the adult mouse thymus. *Immunity* **24**, 53–64 (2006).

14. Kelliher, M. A., Seldin, D. C. & Leder, P. Tal-1 induces T cell acute lymphoblastic leukemia accelerated by casein kinase IIalpha. *EMBO J* **15**, 5160–6 (1996).

15. Draheim, K. M. *et al.* A DNA-binding mutant of TAL1 cooperates with LMO2 to cause T cell leukemia in mice. *Oncogene* **30**, 1252–1260 (2011).

16. Liberzon, A. *et al.* The Molecular Signatures Database Hallmark Gene Set Collection. *Cell Syst* **1**, 417–25 (2015).

17. Koopmans, F. *et al.* SynGO: An Evidence-Based, Expert-Curated Knowledge Base for the Synapse. *Neuron* **103**, 217-234.e4 (2019).

18. Lenth, R. v. The R package lsmeans. *J Stat Softw* **69**, (2016).

19. Benjamini, Yoav ; Hochberg, Y. Controlling the False Discovery Rate - a Practical and Powerful Approach to Multiple Testing. Journal of the Royal Statistical Society Series B-Methodological 1995.pdf. *Journal of the Royal Statistical Society Series B (Methological)* **57**, (1995).

20. Szymanska, B. *et al.* Pharmacokinetic modeling of an induction regimen for in vivo combined testing of novel drugs against pediatric acute lymphoblastic leukemia xenografts. *PLoS One* **7**, e33894 (2012).
